# Characterization of phage resistance and phages capable of intestinal decolonization of carbapenem-resistant *Klebsiella pneumoniae* in mice

**DOI:** 10.1038/s42003-022-03001-y

**Published:** 2022-01-13

**Authors:** Qingqing Fang, Yu Feng, Alan McNally, Zhiyong Zong

**Affiliations:** 1 Center of Infectious Diseases, West China Hospital, Sichuan University, Chengdu, Sichuan China; 2 Division of Infectious Diseases, State Key Laboratory of Biotherapy, Chengdu, Sichuan China; 3 Center for Pathogen Research, Sichuan University, Chengdu, China; 4 Institute of Microbiology and Infection, College of Medical and Dental Sciences, University of Birmingham, Birmingham, UK; 5 Department of Infection Control, West China Hospital, Sichuan University, Chengdu, China

**Keywords:** Bacteriophages, Antimicrobial resistance

## Abstract

Carbapenem-resistant *Klebsiella pneumoniae* (CRKP) has emerged as a severe global health challenge. We isolate and characterize two previously unidentified lytic phages, P24 and P39, with large burst sizes active against ST11 KL64, a major CRKP lineage. P24 and P39 represent species of the genera *Przondovirus* (*Studiervirinae* subfamily) and *Webervirus* (*Drexlerviridae* family), respectively. P24 and P39 together restrain CRKP growth to nearly 8 h. Phage-resistant mutants exhibit reduced capsule production and decreased virulence. Modifications in *mshA* and *wcaJ* encoding capsule polysaccharide synthesis mediate P24 resistance whilst mutations in *epsJ* encoding exopolysaccharide synthesis cause P39 resistance. We test P24 alone and together with P39 for decolonizing CRKP using mouse intestinal colonization models. Bacterial load shed decrease significantly in mice treated with P24 and P39. In conclusion, we report the characterization of two previously unidentified lytic phages against CRKP, revealing phage resistance mechanisms and demonstrating the potential of lytic phages for intestinal decolonization.

## Abstract (web summary)

Fang et al. characterized two previously unidentified phage species that could inhibit growth and decrease virulence of carbapenem-resistant *Klebsiella pneumoniae* (CRKP). They also showed that CRKP develop phage resistance but could still be decolonized in a mouse intestinal colonization model, highlighting phage therapy as potential treatment against drug-resistant pathogens.

## Introduction

*Klebsiella pneumoniae* is a major human pathogen causing a variety of community and hospital-acquired infections such as pneumonia, bacteremia, liver abscess, and meningitis^1^. Carbapenem-resistant *K. pneumoniae* (CRKP) has emerged as a particularly severe challenge for clinical management and public health worldwide as infections due to CRKP are associated with increased mortality rates and elevated healthcare costs^2^. CRKP has been listed as one of the “Critical” priority pathogens that pose the greatest threat to human health by the World Health Organization^3^. Therapeutic options against CRKP are very limited and therefore clinicians and scientists have renewed efforts to identify alternative antibacterial therapies including phages^4^.

Bacteriophages or phages are viruses infecting bacterial hosts. They had been used as antibacterial agents for about 20 years prior to the application of antibiotics in human^4,5^. Due to the introduction of broad-spectrum antibiotics, the research of further application of phages was significantly reduced. Recently, phage therapy has garnered fresh attention as an alternative option for treating infectious diseases^6,7^. However, bacteria can rapidly develop resistance to phages in the course of treatment^7^. To overcome this, cocktails, i.e., the mixtures of multiple phages, are commonly applied to minimize or curb the development of resistance. Discovery of new lytic phages is required to establish expanded libraries that may have potential clinical use.

In this study, we isolated two previously unidentified lytic phages in tandem against CRKP and characterized them by phenotypic and genomic methods. As expected, CRKP isolates rapidly developed phage resistance within 4 h, but the two phages in combination could postpone the emergence of phage resistance to nearly 8 h. We, therefore, analyzed phage-resistant isolates and demonstrate that mutations in the capsule synthesis associated gene *mshA* and glycosyltransferase-encoding gene *epsJ* (involving in the exopolysaccharide synthesis) can mediate phage resistance, which have not been reported before. We also established mouse CRKP colonization models and applied the phages to decolonize CRKP from the intestinal tract of mice. We found the combination of the two phages can significantly reduce CRKP load in the intestinal tract.

## Results

### Recovery of two lytic phages active against ST11 KL64 CRKP

**Fig. 1:**
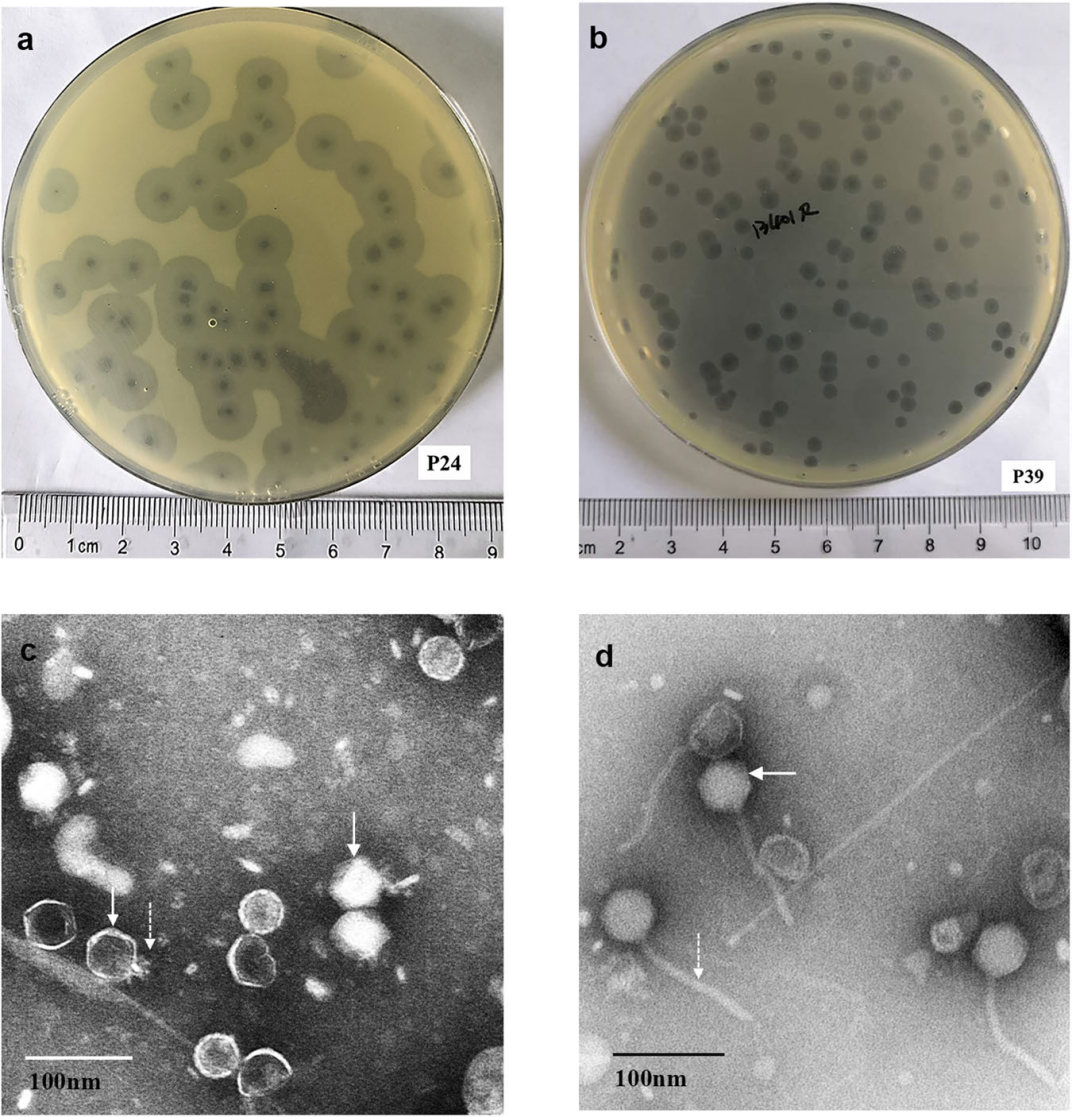
Phage plaques formed on LB agar plates and TEM images of the isolated phages. **a** P24 formed a circular plaque with about 7 mm (diameter) halo surrounding the zone of bacterial clearing; **b** P39 formed a circular plaque with about 2 mm (diameter) halo surrounding the zone of a bacterial clearing. **c** The TEM image of P24 with an ~54 nm icosahedral head (indicated by an arrow) and an ~20 nm tail (indicated by an arrow with a dotted line). **d** The TEM image of P39 with an about 54 nm icosahedral head (indicated by an arrow) and an about 155 nm flexible tail (indicated by an arrow with a dotted line). The 100-nm scale is indicated for **c** and **d**.

A phage able to form a large clear plaque against sequence type 11 (ST11) KL64 (capsule type) CRKP strain B0 on a LB agar plate (Fig. 1a) was isolated and was assigned the name P24. Strain B0 was able to develop resistance to P24 in the bacteria-phage co-culture after 4-h incubation. We, therefore, used a P24-resistant mutant of B0, B1-1, as the host and performed a second round of phage isolation with a sewage sample collected 3 months later than that used in the first round. Another phage, which was able to form a small plaque against B1-1 (Fig. 1b), was isolated and was assigned the name P39. Transmission electron microscopy (TEM) revealed that P24 and P39 exhibited typical features of *Podoviridae* (Fig. 1c) and *Siphoviridae* (Fig. 1d), respectively.

In 20 additional CRKP strains tested, P24 could lyse all the 6 ST11 KL64 CRKP strains tested of different clones but not strains of other STs or other capsular types in spot assays, exhibiting capsular specificity. However, on the plate containing ST11 KL64 strain 090527 in the efficiency of plating (EOP) assay, a turbid zone was observed in the first two dilutions (10^9^ and 10^10^ plaque-forming units [PFU]/mL), but no lysis was present in further dilutions and no individual plaques were observed. P24-susceptible ST11 KL64 strains were not lysed by P39, while their P24-resistant mutants tested were all susceptible to P39 (Supplementary Table 1).

More than 95% of P24 particles were adsorbed to the host strain B0 within 3 min. One-step growth curve revealed that the latent period of P24 was about 20 min and the burst size of P24 was around 85 PFU/cell. By contrast, P39 had a weaker adsorption with only 57.14% adsorbed to B1-1 and a longer latent period (about 25 min), but with a larger burst size, of around 107 PFU/cell (Supplementary Fig. 1a). P24 could not be adsorbed to B1-1, B1-2, and B1-3 with a 0% adsorption. The adsorption of P39 was 0% to B2-1 and B2-3 and was 62.50% to B2-2, which was similar to the 57.14% and 60.00% to B1-1 and B1-2. The susceptibility to different pH conditions showed that both phage particles remained relatively stable within a pH range of 3–11. Incubation at pH 2 caused a 3- and 6-log decrease in the phage titer of P24 and P39 (Supplementary Fig. 1b), respectively. Both phages remained stable at temperatures of 4–50 °C. The titer of P24 and P39 declined by 3- and 1-log at 60 °C, respectively. Both phages were completely inactivated at 70 °C (Supplementary Fig. 1c).

**Fig. 2:**
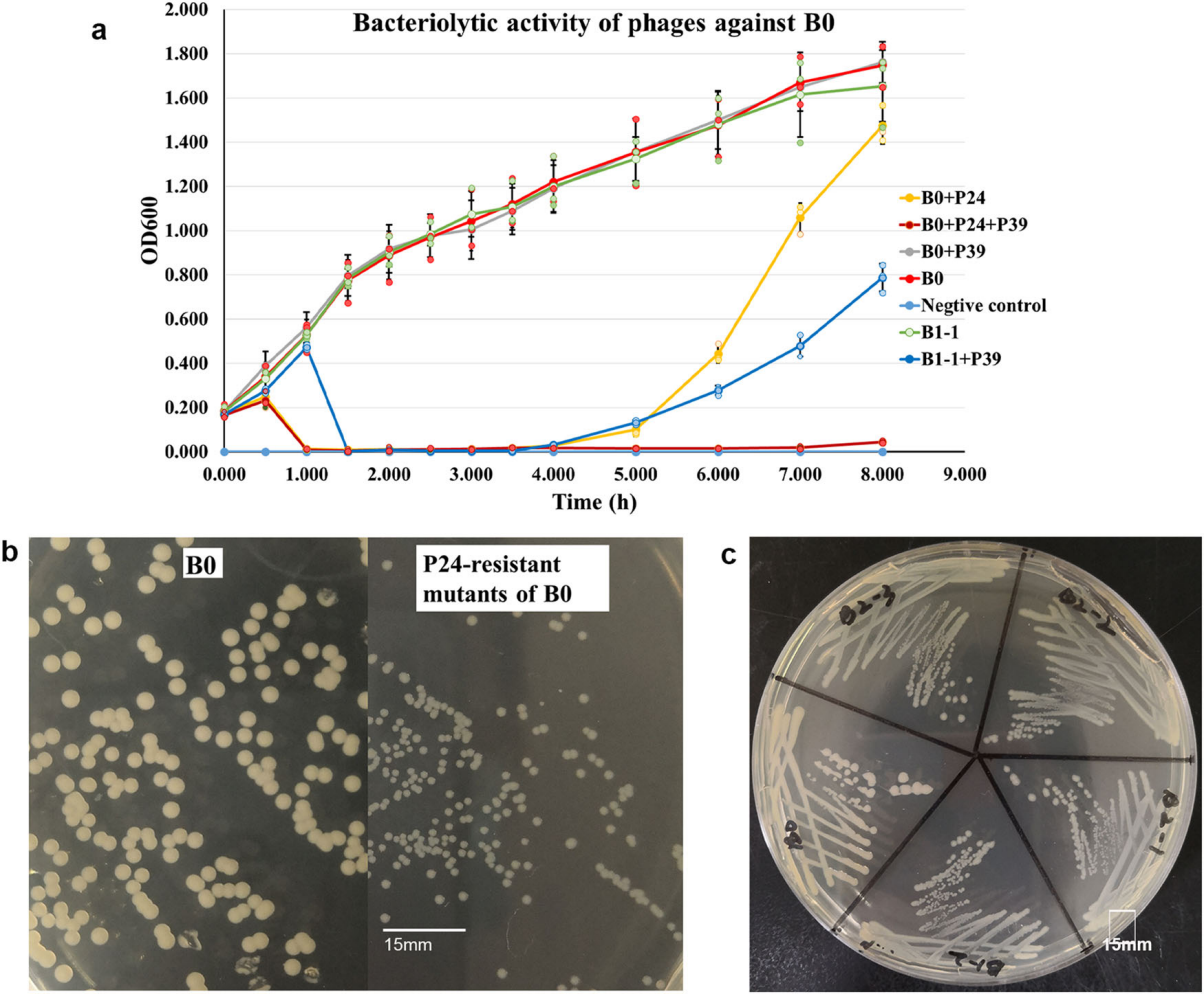
Bacteriolytic activity of phages and morphology switch of phage-resistant mutants. **a** Bacteriolytic activity of phages against strain B0, shown by mean ± SD (*n* = 3). P24 restrained the growth of B0 for 4 h, while the P24 and P39 in combination could prolong the restrain period against B0 to nearly 8 h. **b** Morphology of B0 and its P24-resistant mutants. P24-resistant colonies were consistently transparent and smaller. P24-resistant mutants B1-1, B1-2, and B1-3 were randomly selected from those colonies. **c** Morphology of B0, B1-2, and P39-resistant mutants (B2-1, B2-2, and B2-3). P39-resistant mutants exhibited transparent, small colonies like their parental strain B1-2. The 15 mm scale is indicated for **b** and **c**. The source results are shown in Supplementary Data 1.

P24 completely lysed the host strain B0 of 10^8^ colony forming units (CFU)/mL within 1 h and the initial phage-induced lysis lasted for around 4 h, followed by bacterial regrowth due to the emergence of P24-resistant mutations (Fig. 2a, b). P39-mediated lysis of B1-1 required 1.5 h and lasted for around 4 h with the occurrence of P39-resistant mutants. When P24 and P39 were used in combination, phage-induced lysis continued to nearly 8 h (Fig. 2a). However, mutants with resistance to both P24 and P39 still emerged at 8 h (Fig. 2a). In a phage minimum inhibitory titer test when the initial bacterial concentration was 10^6^ CFU/mL lower than that in the phage bacteriolytic assay, bacteria grew in each of different multiplicity of infection (MOI) tested after exposure to P24 or P39 alone, indicating the emergence of phage-resistant mutants. However, after exposure to a combination of P24 and P39, no bacterial growth was observed at 48 h in the tested MOI.

### The two phages belong to two species and are suitable for therapy

**Fig. 3:**
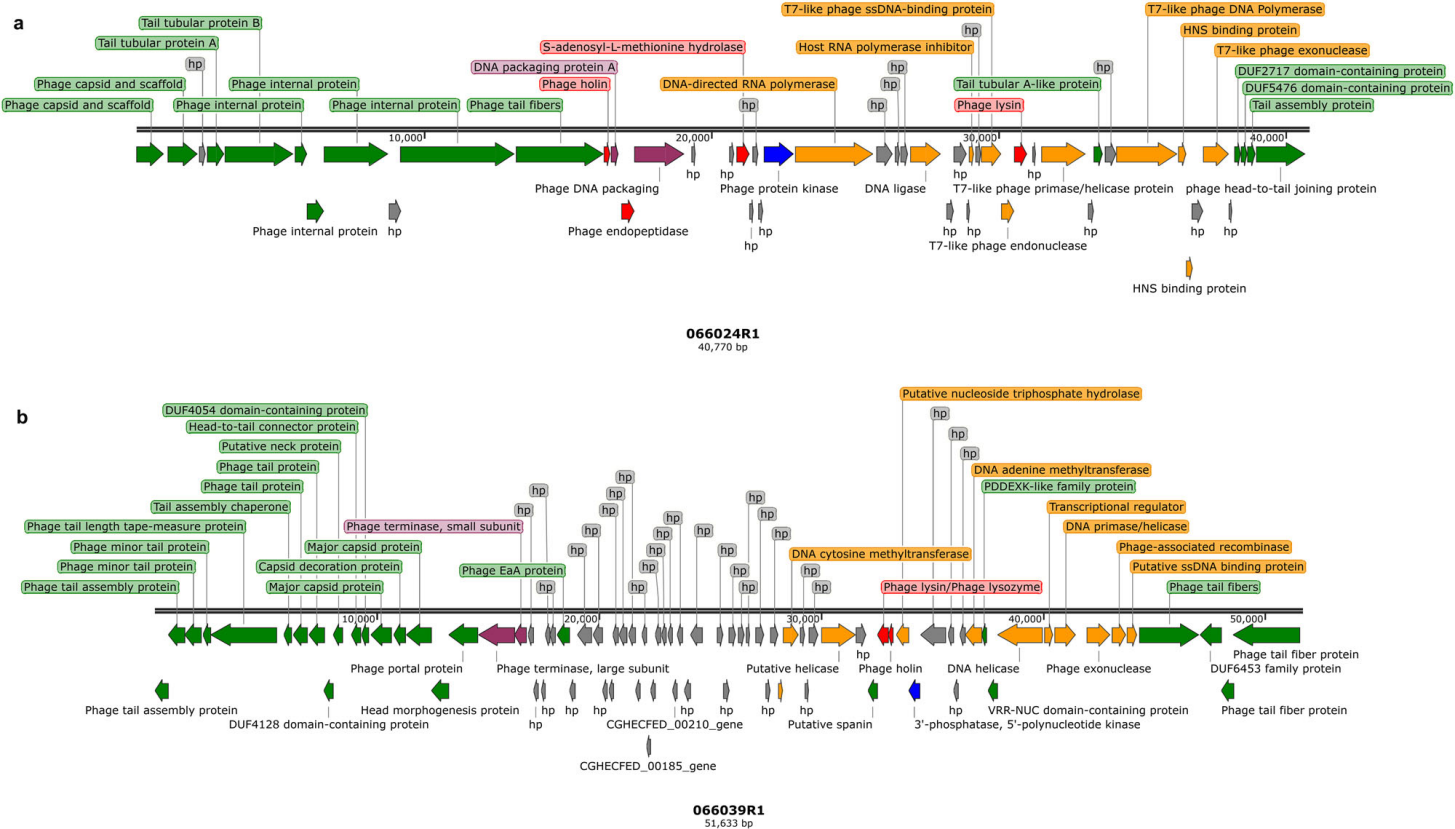
Genomic compositions of P24 and P39. **a** P24. **b** P39. Arrows in different colors represent predicted open reading frames (ORFs) of different functions: red, lysis; gray, hypothetical protein (hp); orange, DNA replication, and transcription; green, phage structure; purple, DNA packaging; and blue, metabolism function. The genome maps were generated using SnapGene (https://www.snapgene.com/).

P24 has a double-stranded DNA (dsDNA) genome of 40,770 bp with 50 predicted coding sequences (CDS) (Fig. 3a and Supplementary Tables 2–4). When compared to all sequenced phages on NCBI, P24 shared the highest nucleotide identity (96.00% coverage and 95.73% identity) with Klebsiella phage vB_Kp1 (accession no. KT367885.1, Supplementary Table 5), which belongs to the genus *Przondovirus* of the recently established family *Autographiviridae* (*Caudovirales* order), previously known as a subfamily of the family *Podoviridae*^8^. P39 has a dsDNA genome of 51,633 bp with 82 predicted CDS (Fig. 3b and Supplementary Tables 2, 6, 7), with the closest identity to *Klebsiella* phage vB_KpnS_KpV522 (accession no. NC_047784.1), which belongs to the genus *Webervirus* within the family *Drexlerviridae*^8^, previously part of the family *Siphoviridae* (*Caudovirales* order), with 87.00% coverage and 97.10% identity (Supplementary Table 8). The main species demarcation criterion for bacterial and archaeal viruses is set at the genomic similarity of 95% according to the International Committee on Taxonomy of Viruses (ICTV)^8^. P24 and P39 had 91.90% and 84.48% overall DNA sequence similarity with their closest taxonomic relatives, vB_Kp1 and vB_KpnS_Domnhall, respectively (Supplementary Tables 5 and 8). Phylogenetic analysis based on amino acid sequences of the DNA polymerase or those of the major capsid protein also revealed that P24 and P39 belonged to the genus *Przondovirus* and *Webervirus*, respectively (Supplementary Fig. 2).

P24 and P39 formed plaques with a halo surrounding the zone of bacterial clearing (Fig. 1a, b). Phage plaque halo is typically associated with phage depolymerases, which appear as a tail spike or fiber proteins in phages of the families *Podoviridae*, *Autographiviridae*, *Siphoviridae*, and *Drexlerviridae*^9,10^. P24 encodes a tail fiber protein (Supplementary Table 3), which had 97.54% identity and 100% coverage with the depolymerase (a tail fiber protein, called ORF42, accession no. YP_009797016.1) of *Klebsiella* phage SH-KP152410^11^. The tail fiber protein of P39 (Supplementary Table 6) had 83.61% identity and 80% coverage with the depolymerase (a tail fiber protein, called Gp50, accession no. YP_009226010.1) of *Klebsiella* phage KP36^12^. This suggests that the tail fiber proteins of P24 and P39 are likely phage depolymerases.

Sequence analysis of the two phages also showed that both encoded phage holin and endolysin protein and did not contain any lysogenic factors, indicating that they were related to strictly lytic phages as suggested previously^13^. Neither phage carried genes encoding antimicrobial resistance, toxins, or other virulence factors. Therefore, both phages met the recommended criteria for therapeutic phage selection^6^.

### Phage-resistant mutants had no alterations in antimicrobial susceptibility

We observed rapid switching of the colony morphology from mucoid to dry for strain B0 after exposure to P24. In the absence of P24, wild-type strain B0 displayed mucoid colony morphology. After P24 treatment, phage-resistant colonies were consistently dry, transparent, and relatively smaller (Fig. 2b). We randomly selected three phage-resistant colonies, which were assigned B1-1, B1-2, and B1-3, for further studies. Previous studies^5,14^ have reported that phage-resistant mutants may have altered antimicrobial susceptibilities as a trade-off. As such, we performed antimicrobial susceptibility testing for the three phage-resistant mutants. Minimum inhibitory concentrations (MICs) of trimethoprim/sulfamethoxazole against B1-1 and B1-3 were <0.5/19 μg/mL, significantly lower than the 128/2432 or 64/1216 μg/mL against B0 and B1-2 (Table 1). Otherwise, there were no significant (≥4-fold) differences between MICs of all other tested agents including ceftazidime, imipenem, and meropenem (Table 1).

**Table 1:** MICs (μg/mL) of antimicrobial agents against strain B0, P24-resistant mutants (B1-1, B1-2, and B1-3), and P39-resistant mutants (B2-1, B2-2, and B2-3).

Antimicrobial agents	B0	B1-1	B1-2	B1-3	B2-1	B2-2	B2-3
Amikacin	**>512**	**>512**	**>512**	**>512**	**>512**	**>512**	**>512**
Aztreonam	**>512**	**>512**	**>512**	**>512**	**>512**	**>512**	**>512**
Ceftazidime	**128**	**64**	**128**	**128**	**64**	**128**	**128**
Ceftazidime/avibactam	2/4	1/4	1/4	1/4	1/4	1/4	1/4
Erythromycin	**>512**	**>512**	**>512**	**>512**	**>512**	**>512**	**>512**
Fosfomycin	**>512**	**>512**	**>512**	**>512**	**>512**	**>512**	**>512**
Imipenem	**64**	**64**	**64**	**64**	**64**	**64**	**64**
Levofloxacin	**32**	**32**	**32**	**32**	**32**	**32**	**32**
Meropenem	**128**	**128**	**128**	**128**	**128**	**128**	**128**
Piperacillin/tazobactam	**>128/4**	**>128/4**	**>128/4**	**>128/4**	**>128/4**	**>128/4**	**>128/4**
Colistin	1	2	1	1	1	1	1
Tigecycline	2	1	2	1	2	2	2
Trimethoprim/sulfamethoxazole	**128/2432**	<0.5/9.5	**64/1216**	<0.5/9.5	**64/1216**	**64/1216**	**64/1216**

Resistance is highlighted in bold.

The difference of susceptibility in B1-1 and B1-3 compared to B0 is underlined.

By comparing genome sequences of B1-1 and B1-3 with those of B0 and B1-2, we found that a plasmid of the IncFIB(K) replicon type carrying a trimethoprim-resistant gene *dfrA12*, assigned pB1-3, was absent from B1-1 and B1-3. However, there was no significant difference in the frequency of plasmid loss between B0 with and without exposure to P24 (25.0% vs 20.8%, *P* = 0.579) in the 5-h culture. This suggests that the plasmid loss was not a direct result of infection with P24 but was due to the unstable nature of the plasmid.

After exposure to P39, P39-resistant mutants also emerged from P24-resistant B1-2. We also randomly selected three P39-resistant colonies, assigned B2-1, B2-2, and B2-3 for further study. These three P39-resistant mutants also remained resistant to P24 and exhibited transparent, small colonies like their parental strain B1-2 (Fig. 2c). We, therefore, tested the antimicrobial susceptibility of the three P39-resistant mutants. There were no significant (≥4-fold) differences between MICs of all tested antimicrobial agents against P39-resistant mutants and those against P39-susceptible B1-2 (Table 1).

### Phage-resistant mutants had lower virulence and showed discrepant resistance to serum

**Fig. 4:**
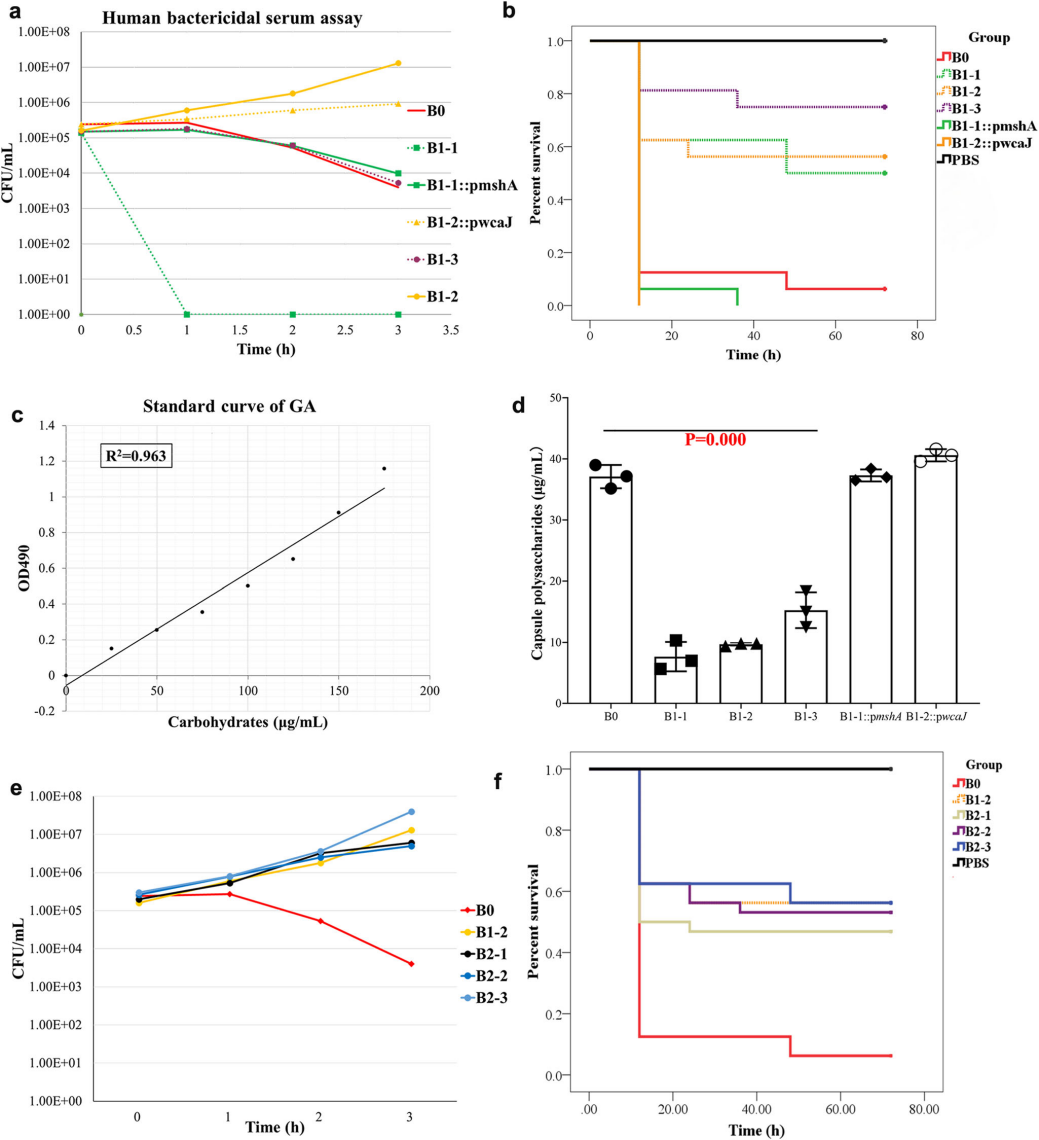
Serum killing, virulence, and capsule contents of strain B0, P24-resistant mutants, and P39-resistant mutants. **a** Human bactericidal serum assays. After mixing with serum for 1 h, >99% of B1-1 was killed, while almost none of B0 and B1-3 were killed, and B1-2 showed increased resistance to serum. Serum resistance was completely and partially restored for B1-1::p*mshA* and B1-2::p*wcaJ*, respectively. **b** *G. mellonella* infection models. The survival rate of larvae infected by P24-resistant mutants B1-1, B1-2, and B1-3 was significantly higher than that by strain B0 (50.00%, 56.25%, 75.00% vs 6.25%; *P* = 0.003, 0.002 and 0.000, respectively). B1-1::p*mshA* and B1-2::p*wcaJ* restored virulence in the *G. mellonella* larvae model with a 0% survival rate. **c** Standard curve of glucuronic acid (GA). This curve was established using a colorimetric assay. **d** Production of capsule polysaccharides (CPS). The CPS production of phage-resistant mutants B1-1, B1-2, and B1-3 significant decreased (mean ± SD, 7.67 ± 2.40, 9.69 ± 0.25, 15.28 ± 2.93 vs 37.11 ± 1.92 μg/mL; *P* = 0.000, 0.000 and 0.000, respectively) compared with that of B0. In contrast, the CPS production of B1-1::p*mshA* and B1-2::p*wcaJ* were restored (37.11 ± 0.98 and 40.60 ± 1.00 μg/mL, respectively) to the level of B0. Different colors represent different strains and complementary strains. Data are shown mean ± SD (*n*  =  3). **e** After mixing with serum for 1 h, B1-2 showed increased resistance to serum. The three P39-resistant mutants, B2-1, B2-2, and B2-3, showed increased resistance to serum-like B1-2 with counts increased in serum at 1 h and further increased to 10^7^ CFU/mL at 3 h. **f** The survival rate of larvae infected by P39-resistant mutants B2-1, B2-2, and B2-3 was similar to that by B1-2 (46.87%, 53.13%, and 56.25% vs 56.25%; *P* = 0.540, 0.838 and 1.000, respectively). The source results are shown in Supplementary Data 2.

Previous studies^15,16^ have reported that phage-resistant mutants are more likely to be killed by serum. We, therefore, performed serum bactericidal assays for B0 and its phage P24-resistant mutants B1-1, B1-2, and B1-3. After mixing with serum for 1 h, >99% of B1-1 was killed, while almost none of B0 and B1-3 were killed (Fig. 4a). Conversely, B1-2 showed increased resistance to serum as the amount of B1-2 in serum slightly increased at 1 h and further increased to 10^7^ CFU/mL at 3 h in contrast to the decrease of B0 from 10^6^ CFU/mL at 1 h to 10^3^ CFU/mL at 3 h (Fig. 4a).

We found that the survival rate of *G. mellonella* larvae infected by P24-resistant mutants B1-1, B1-2, and B1-3 (Fig. 4b) was significantly higher than that by strain B0 (50.00%, 56.25%, and 75.00% vs 6.25%; *P* = 0.003, 0.002, and 0.0000, respectively). This indicates that the phage-resistant mutants had lower virulence, which may be a trade-off for bacteria to develop phage resistance. Standard curve of glucuronic acid revealed that the relationship between absorbance and glucuronic acid content was linear (Fig. 4c). As capsules are major determinants of virulence, we measured the capsule production of phage-resistant mutants B1-1, B1-2, and B1-3 and found significantly decreased CPS (capsule polysaccharides; mean ± standard deviation [SD], 7.67 ± 2.40, 9.69 ± 0.25, and 15.28 ± 2.93 vs 37.11 ± 1.92 μg/mL; *P* = 0.000, 0.000, and 0.000, respectively) in the three phage-resistant mutants (Fig. 4d).

We also performed serum bactericidal assays and examined the virulence of P39-resistant mutants (B2-1, B2-2, and B2-3). The three mutants showed increased resistance to serum-like B1-2 with increased counts in serum at 1 h and a further increase to 10^7^ CFU/mL at 3 h (Fig. 4e). Like its parental P24-resistant strain B1-2, the three P39-resistant mutants had lower virulence than B0 with higher survival rates for infected *G. mellonella* larvae. The virulence of the three mutants was not significantly different from B1-2 with similar survival rates (46.87%, 53.13%, and 56.25% vs 56.25%; *P* = 0.540, 0.838 and 1.000, respectively) (Fig. 4f), but significantly different from B0 with higher survival rates (vs 6.25%; *P* = 0.008, 0.002, and 0.001, respectively).

### Modifications of CPS genes and the exopolysaccharide (EPS) gene result in phage resistance

**Fig. 5:**
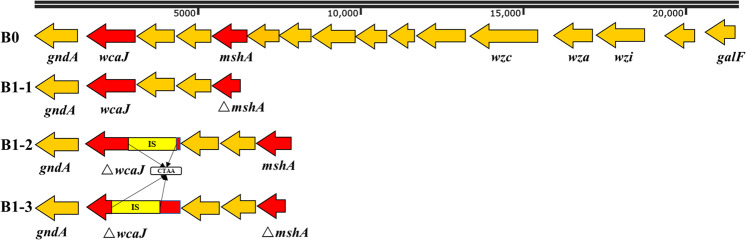
The *cps* gene cluster in strain B0 and the interrupted *wcaJ* gene in P24-resistant B1-2 and B1-3. The gene cluster contains *galF* (encoding a UDP-glucose pyrophosphorylase), *gndA* (encoding a gluconate-6-phosphate dehydrogenase), *wcaJ* (encoding a glycosyltransferase), *wza* (encoding a capsule polysaccharide export protein), *wzc* (encoding a tyrosine-protein kinase), and *wzi* (related to capsule surface assembly)^60^. *wcaJ* was interrupted by IS*Kpn26* at the 35^th^ nucleotide position in B1-2 and at the 409th nucleotide position in B1-3 with the 4-bp direct target repeats (CTAA) characteristic of the insertion of IS*Kpn26*.

Compared with strain B0, 9 single nucleotide polymorphisms (SNPs) were identified in both B1-1 and B1-3 (Table 2). The 9 SNPs are identical between B1-1 and B1-3 and there were no SNP differences between these two mutants. There was an insertion of nucleotide G in the D-inositol-3-phosphate glycosyltransferase-encoding *mshA* gene, resulting in a frameshift, while other SNPs were nonsense mutations or missense mutations in genes encoding hypothetical proteins of unknown function (Table 2). By contrast, there was no SNP in B1-2 compared with B0. However, the glycosyltransferase-encoding *wcaJ* gene was interrupted by insertion sequence IS*Kpn26* at the 35^th^ nucleotide position (numbered from the start codon) in B1-2 and at the 409th nucleotide position in B1-3 with the 4-bp direct target repeats characteristic of the insertion of IS*Kpn26* (Fig. 5).

**Table 2:** SNPs in P24-resistant mutants B1-1 and B1-3 compared with their parental strain B0.

Contig	Position	Type	B0	B1-1/B1-3	Region	Effect	Gene	Product
contig00017	15828	ins	G	GC	CDS	frameshift_variant c.46dupG p.Ala16fs	*mshA*	d-inositol-3-phosphate glycosyltransferase
contig00035	14859	snp	C	T	Non-coding regions			
contig00087	1430	snp	T	C	Non-coding regions			
contig00088	96	snp	G	C	Non-coding regions			
contig00088	101	snp	G	A	Non-coding regions			
contig00088	1247	snp	A	T	Non-coding regions			
contig00088	1446	snp	T	C	CDS	missense_variant c.73A>G p.Ile25Val		Hypothetical protein
contig00122	96	snp	G	C	Non-coding regions			
contig00122	221	snp	G	A	Non-coding regions

**Fig. 6:**
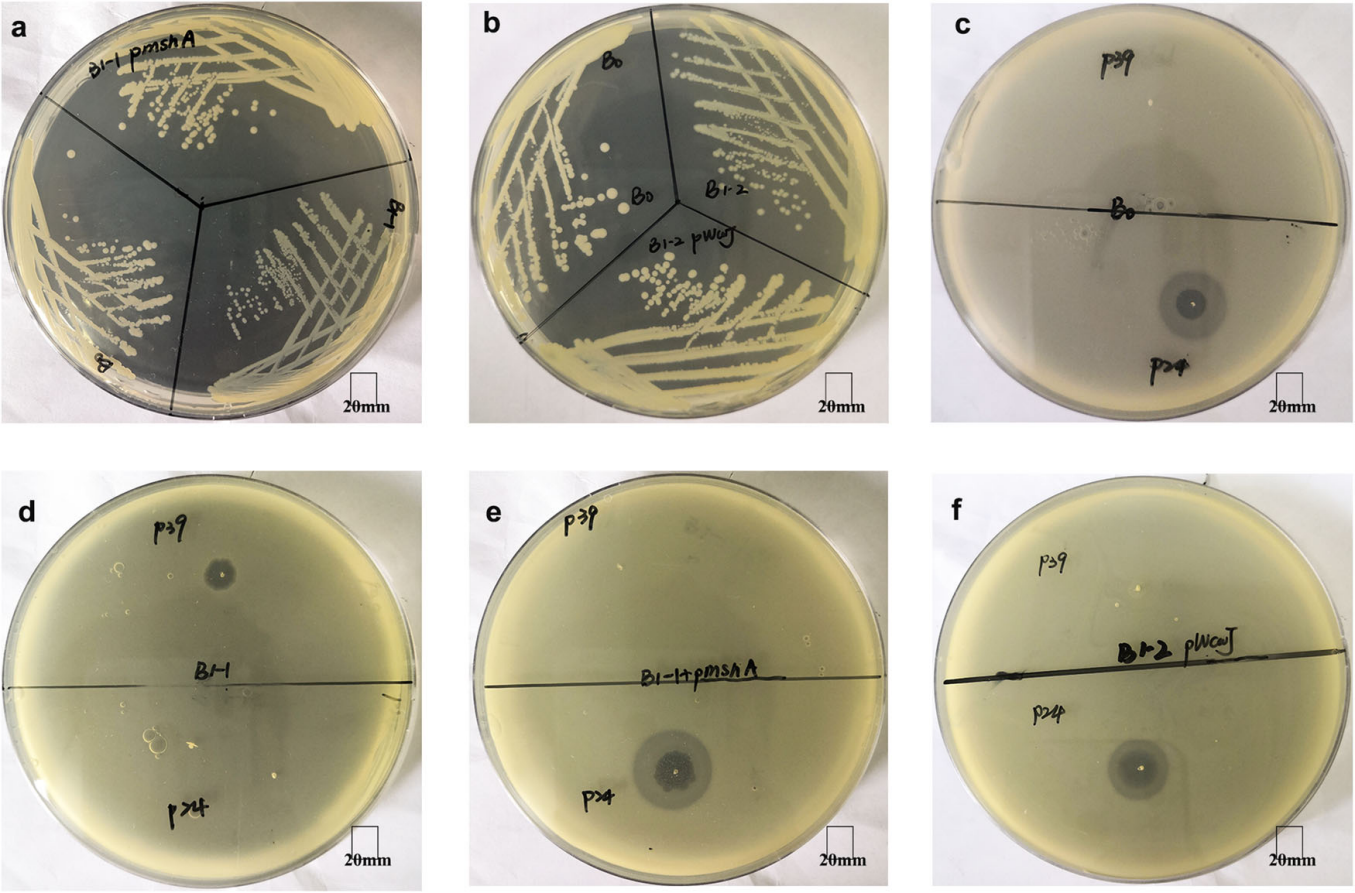
Colony appearance of strain B0, P24-resistant mutants and transformants, and spot test assays. **a** Colony appearances of B1-1 and B1-1::p*mshA* compared with B0. **b** Colony appearances of B1-2 and B1-2::p*wcaJ* compared with B0. **c**–**f** Spot test assays of P24 and P39 on B0, B1-1, B1-1::p*mshA,* and B1-2::p*wcaJ*, respectively. P24 formed a clear large clearing zone with 13–15 mm (diameter) halos and a 7–8 mm hole in the middle of the zone in semi-solid LB plates containing strain B0, B1-1::p*mshA* and B1-2::p*wcaJ*. P39 formed a 7 mm clearing zone without a halo in plates containing P24-resistant mutant B1-1. The 20 mm scale is indicated.

As mentioned above, phage P24-resistant mutants exhibited smaller colony morphology (Fig. 6a, b) and lost susceptibility to phage P24 (Fig. 6c, d). To examine whether the frameshift of *mshA* in mutant B1-1 causes these changes and results in attenuated virulence, we cloned the wild-type *mshA* from strain B0, assigned *mshA*_B0, into P24-resistant mutant B1-1. A transformant of B1-1 containing *mshA*_B0, assigned B1-1::p*mshA*, was obtained and the presence of *mshA*_B0 was confirmed by PCR and subsequent Sanger sequencing. The colony morphology was restored to the mucoid appearance for B1-1::p*mshA* (Fig. 6a). Spot tests showed that B1-1::p*mshA* became susceptible to P24 (Fig. 6e) but lost susceptibility to P39 as expected. In serum bactericidal assay, almost no B1-1::p*mshA* cells were killed in 1 h (Fig. 4a), suggesting that *mshA* also mediates resistance to serum and the capsule protects bacteria from killing by serum.

As mentioned above, B1-2 had no *mshA* mutations but had an interrupted *wcaJ*. We, therefore, cloned the wild-type *wcaJ* gene from strain B0, assigned *wcaJ_*B0, into B1-2. We obtained B1-2 containing *wcaJ*_B0, assigned B1-2::p*wcaJ*, with the presence of *wcaJ*_B0 being confirmed by PCR and subsequent Sanger sequencing. As expected, B1-2::p*wcaJ* restored susceptibility to P24 (Fig. 6f) and the mucoid appearance (Fig. 6b) but became resistant to P39 (Fig. 6f). In addition, both B1-1::p*mshA* and B1-2::p*wcaJ* restored CPS levels (mean ± SD; 37.11 ± 0.98 and 40.60 ± 1.00 μg/mL, respectively) (Fig. 4d) and virulence in *G. mellonella* larvae with a 0% survival rate (Fig. 4b). This confirms that the two genes are involved in the capsule production and virulence of CRKP. In contrast to the 95% adsorption of P24 to strain B0, adsorption to P24-resistant mutants B1-1, B1-2, and B1-3 was 0%. This indicates that the decreased CPS prevents bacterial strains from absorbing phage P24 and therefore results in phage resistance.

**Fig. 7:**
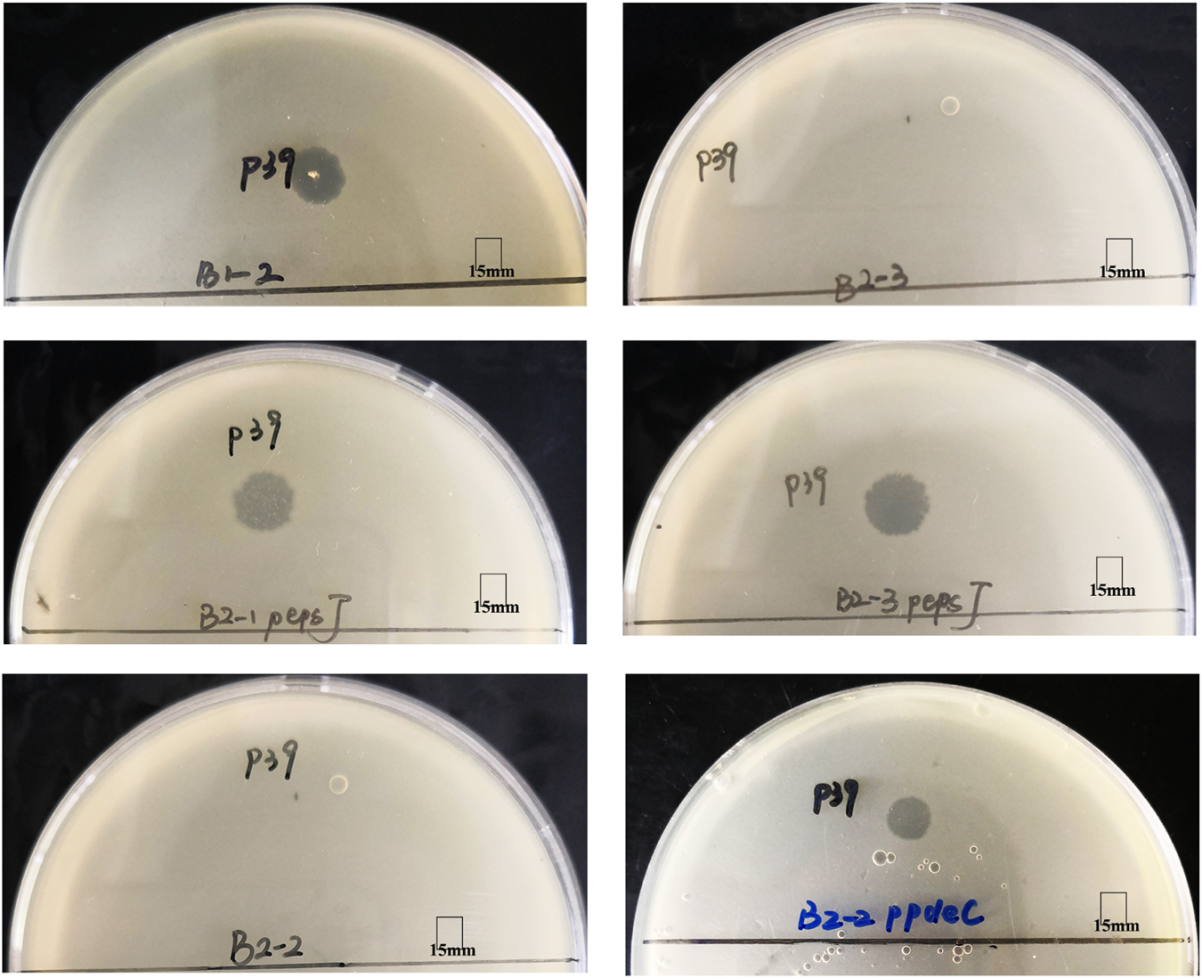
Phage P39 sensitivity test assay of B1-2, a P39-resistant mutant, and three transformants. Spot test assays of P39 on B1-2, P39-resistant mutant B2-3 and transformants B2-1::p*epsJ*, B2-3::p*epsJ*, and B2-2::p*pdeC*. All three transformants restored susceptibility to P39. P39 formed around 7 mm clearing zone without a halo against B1-2 and the three transformants. The 15 mm scale is indicated.

Compared with P24-resistant mutant B1-2, there were 8, 8, and two SNPs identified in P24- and P39-resistant mutants B2-1, B2-2, and B2-3, respectively (Supplementary Table 9). A stop-gained variant Gln178* was found in the glycosyltransferase-encoding *epsJ* gene in B2-1, while the *epsJ* gene was interrupted by insertion sequence IS*Kpn14* at the 779th nucleotide position (numbered from the start codon) in B2-3. We, therefore, cloned the wild-type *epsJ* gene from strain B1-2 into B2-1 and B2-3. Both transformants, B2-1::p*epsJ* and B2-3::p*epsJ*, restored susceptibility to P39 (Fig. 7) but not to P24. In addition, a missense variant Asp70Tyr of the cyclic di-GMP phosphodiesterase PdeC was present in B2-2. Mutations of the cyclic di-GMP pathway have been previously found to mediate phage resistance in bacteria^17,18^. We also cloned the wild-type *pdeC* from P39-susceptible B1-2 into its P39-resistant mutant B2-2 containing a SNP in *pdeC*. The transformant B2-2::p*pdeC* restored susceptibility to P39 but not to P24 (Fig. 7), confirming the involvement of this mutation of *pdeC* in phage resistance.

### Combination of P24 and P39 reduced bacteria shed count from mice

Prior to experimentation, we confirmed that there were no phages capable of lysing strain B0 in the intestinal tract of all mice used in the study by the aforementioned phage isolation method. After administration by oral water containing P24 or by gavage, P24 was not detected in the feces of B0-colonized mice. When P24 was administered by enema, its concentration in feces fluctuated between 10^2^ and 10^7^ PFU/g. After administration, the concentration of P24 in the intestinal tract dropped sharply and became undetectable the next day. Therefore, P24 was administered by enema for further studies. Following administration via oral gavage, the concentration of P39 in feces fluctuated between 10^2^ and 10^6^ PFU/g. When P39 was added to the daily drinking water of mice, its concentration in feces was maintained at 10^7^ to 10^8^ PFU/g. After administration, the concentration of P39 in the intestinal tract dropped to 10^3^–10^5^ PFU/g in the next day and further declined to the lower limit of detection on day three. Healthy mice treated with P24 by enema and oral water containing P39 in combination did not develop adverse reactions such as diarrhea, disturbance of consciousness, or death.

**Fig. 8:**
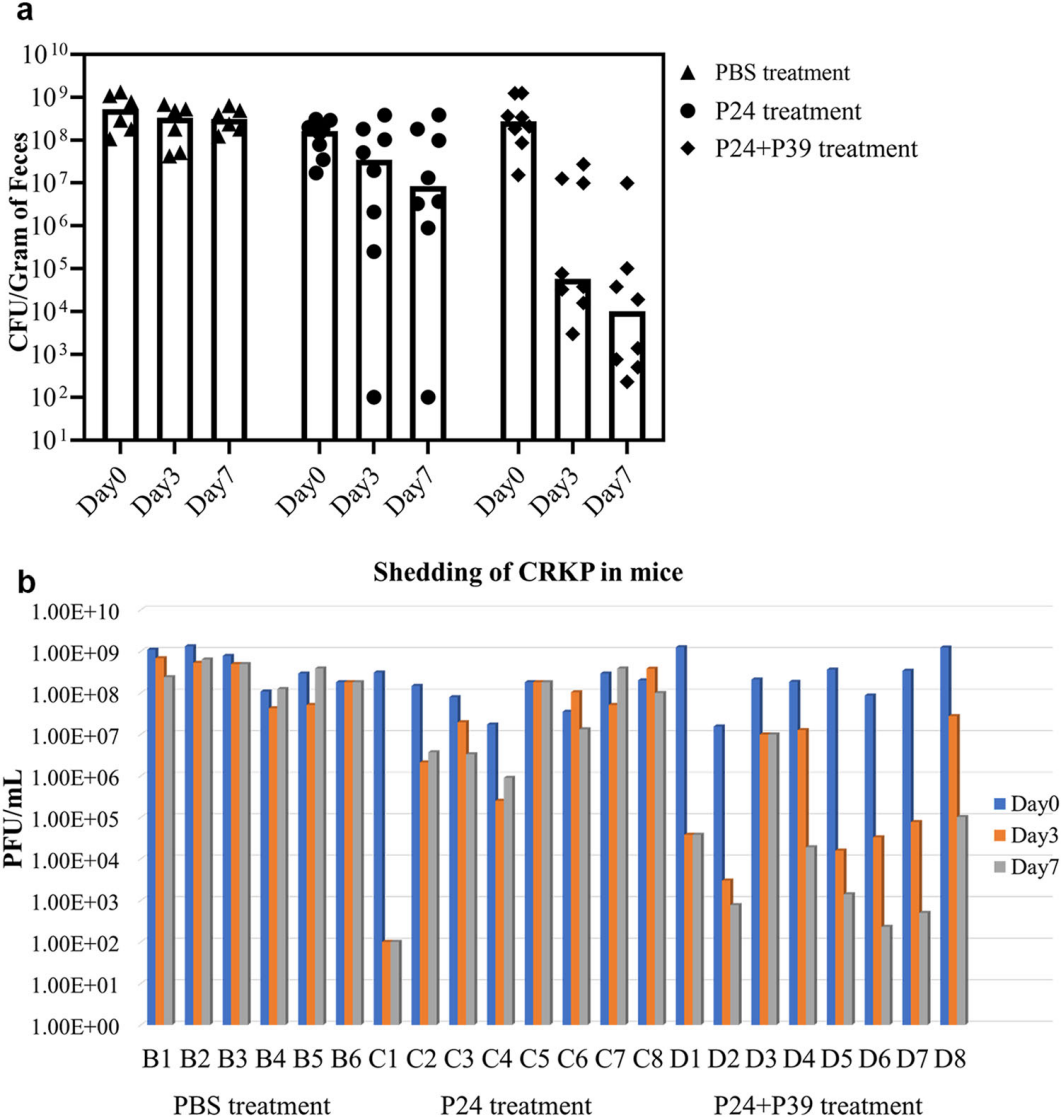
Shedding of strain B0 in mice. **a** Bar graphs show the median colonization density of strain B0 in feces at different time points from mice after different treatment. Each data point represents colonization density in a specific treatment from individual mice. The detection limit was 10^2^ CFU/g. **b** Shedding of CRKP in an individual mouse of each group. B/C/D represents the number of mice in each group. The source results are shown in Supplementary Data 3.

Under a pre-treatment of continuous administration of drinking water containing meropenem, strain B0 was able to stably colonize in the intestinal tract of all mice and was shed in feces at levels around 10^8^ CFU/g during the entire 10-day observation period in the control group administrated with phosphate-buffered saline (PBS). After stable shedding of strain B0 for the first three days, P24 was administered to 8 mice. Among the 8 P24-treated mice, the shedding of strain B0 in 7 mice decreased by 1–2-log on day one but P24-resistant mutants were detected on day three and persisted for the next four days at 10^5^ to 10^8^ CFU/g. Whole-genome sequencing revealed that like B1-2, all of the three randomly selected P24-resistant mutants from mouse feces (B1-4, B1-5, and B1-6) also had an interruption of *wcaJ* by IS*Kpn26* at the 1146^th^ nucleotide position. We randomly selected another 20 P24-resistant mutants from mouse feces and amplified their *wcaJ* and *mshA* genes by PCR. Sanger sequencing of PCR amplicons revealed that all 20 P24-resistant mutants had *wcaJ* interruption by insertion sequence IS*903B*, IS*Kpn14*, or IS*Kpn26* at the 1146th nucleotide position or 12 additional positions (Supplementary Table 10). Four of the 20 mutants also had a deletion of nucleotide A at the 50th nucleotide position of *mshA* (Supplementary Table 10). In the remaining one mouse, strain B0 decreased from 10^8^ to 10^2^ CFU/g on day three and persisted for the next 4 days at the same titer (Fig. 8a, b).

By contrast, in another 8 mice administered with both P24 and P39, the shedding of strain B0 in 7 mice decreased by 4- to 6-log compared with the initial shedding count by day 7, with counts ranging between 10^2^ and 10^5^ CFU/g (Fig. 8b). In the remaining mouse, smaller and dry colonies resistant to both P24 and P39 appeared on culture plates on day three, and the shedding of strain B0 or its mutants remained at 10^7^ CFU/g feces up to day 7, only slightly lower than the 10^8^ CFU/g observed in the control group (Fig. 8b).

## Discussion

In this study, we isolated and characterized two previously unidentified lytic phages against ST11 KL64 CRKP isolates. The two phages represent two species belonging to the genus *Przondovirus* of the family *Autographiviridae* and the genus *Webervirus* of the family *Drexlerviridae*, respectively. Both phages exhibited stability at various temperatures and pH. Host range analysis revealed that both phages were only able to lyse ST11 KL64 *K. pneumoniae*, which is the dominant CRKP type in China^19,20^. The capsule specificity also suggests that the use of the two phages is unlikely to disturb human microflora. The two phages in this study have good self-replication capability with a large burst size, which is different from many previously reported phages recovered from *K. pneumoniae* including both CRKP and carbapenem-susceptible isolates^21,22^. Phage P24 was able to reduce the number of bacteria to a minimum within 1 h, but this inhibitory effect lasted only 4 h until resistant mutants arose. Such a short life of inhibition by P24 is consistent with findings in previous reports^23,24^. When used in combination with P39, the inhibitory effect was doubled to nearly 8 h, suggesting a synergistic effect between the two phages to postpone the emergence of resistant mutants. The second phage, P39, was isolated with a mutant resistant to the first phage, P24, as the host strain. This suggests that such a layered strategy to isolate lytic phages in tandem is a practical approach for isolating phages to construct effective cocktails for therapy. Multiple rounds of phage isolation using mutants that developed resistance to phages isolated in previous rounds may be required to generate a cocktail able to maximize killing the target bacterial strain. When the initial concentration of bacteria was 10^8^ CFU/mL, phage-resistant mutants always occurred regardless of the MOI. However, when the initial number of bacteria exposed to the phage combination was decreased to 10^6^ CFU/mL, the combination of P24 and P39 could completely kill the bacteria without the emergence of resistant mutants at 48 h in vitro at any MOI. We, therefore, speculated that the incidence of phage-resistant mutants in the bacterial population is less than 1 in 10^6^. This indicates that the combination of the two phages may be effective in an infection site with lower bacterial loads.

Resistant mutants do emerge when treated with phages in vitro and in vivo. A few studies have found that phage-resistant mutants incur a significant fitness cost such as reduced resistance to serum killing and restored antimicrobial susceptibility as a trade-off of developing phage resistance^7,25–27^. In this study, we found that phage-resistant mutants exhibited a smaller colony appearance, reduced capsule production, attenuated virulence, and compromised resistance to serum killing. These features are consistent with the previous reports^15,27,28^ and are beneficial to the ability of the human immune system to eliminate phage-resistant mutants. A single P24-resistant mutant (B1-2) showed increased resistance to the serum, suggesting that not all phage-resistant mutants were more vulnerable for serum killing. Fortunately, the virulence of all resistant mutants in this study was clearly attenuated, which again would be beneficial for managing any corresponding infection.

CRKP colonization in the intestinal tract usually predisposes CRKP infections^29,30^. The application of lytic phages to treat CRKP infections in mice has been well documented in the literature such as reference^31–33^. By contrast, studies of phages to decolonize CRKP from the intestinal tract are scarce. Decolonization by phages appears to be more challenging than phage therapy for infections. In infections, the human immune system responds to inflammation driven by pathogens and therefore participates in controlling the infection. Since phage-resistant mutants are usually attenuated in virulence, it is likely that they would be even more easily cleared by a pro-inflammatory host response. By contrast, bacterial colonization does not induce protective immune responses. Given the large numbers of bacterial cells in the intestinal tract, the development of phage-resistant mutants should happen quickly in the absence of a pro-inflammatory response to help clear them. We established a murine model of intestinal colonization with CRKP to study the effect of decolonization by administrating phages. As expected, CRKP strain B0 quickly developed resistance to P24 in vivo and after administration of P24, the number of bacteria in mice feces only decreased slightly. However, when P24 and P39 were administered in combination, the number of bacteria in mouse feces further reduced, almost to the lower limit of detection of 10^2^ CFU/g feces. These results are highly indicative of the fact that phages can be used for the decolonization of CRKP from the intestinal tract and that phage-resistant mutations incur a significant colonization defect even in the absence of a pro-inflammatory immune response. As such phage combinations could have a significant impact on nosocomial transmission and spread of CRKP.

We had attempted to administer P24 by oral or gavage but no P24 could be detected in mouse feces and phage titer in the intestinal tract did not increase due to bacteriolytic effects as expected in B0-colonized mice. Therefore, continuous administration of phages was needed. It is possible that the phage produced by self-proliferation is cleared by substances in the intestinal tract. Therefore, microcapsules may be required to protect phage particles from degradation by digestive enzymes in the gastrointestinal tract, to reach a certain concentration in the intestine. Further studies are warranted.

We have shown that modifications of *mshA* and *wcaJ* mediated resistance to phage P24. Both genes are part of the CPS-encoding *cps* module (Fig. 5). WcaJ is a membrane protein functioning as a glycosyltransferase initiating the synthesis of colanic acid^34^, which is the major CPS in the *Enterobacteriaceae*^35^. Previous studies have shown that disruption of *wcaJ* results in the absence of colanic acid and therefore renders a non-mucoid appearance^36^ and phage resistance^37,38^. By contrast, the link between *mshA* and phage resistance has not been reported before. The *mshA* gene is located upstream of *wcaJ* and encodes a glycosyltransferase family 4 protein. Interestingly, when the *mshA* gene of strain B0, which is P24-susceptible but P39-resistant, was introduced into its P24-resistant P39-susceptible mutant B1-1, the generated B1-1 transformant lost its susceptibility to P39, providing insights into the resistance mechanism for this phage. Using a conventional colorimetric assay, we found that phage-resistant mutants showed a significant decrease in capsule formation. A previous study^39^ has reported that some CPSs may sterically mask surface receptors to block the binding of certain phages, while other CPSs may serve as obligate receptors of other phages. In our study, the adsorption of P24 to B1-1, B1-2, and B1-3 was 0% even after prolonged incubation time. This suggests that phage P24 recognizes and adsorbs to certain CPS residues. The CPS content of P24-resistant mutants was reduced compared to the parental P24-susceptible strain. Therefore, the modifications of *mshA* and *wcaJ* could alter CPSs synthesized and the altered CPSs may be an obstacle to P24. Epidemiological studies have revealed that the central region of the *cps* gene clusters in *K. pneumoniae* is highly divergent^40^. Both *wcaJ* and *mshA* are located in the *cps* gene variable region^40^. Our study confirmed that the genetic variation of the sugar moiety and polysaccharide linkage generate the diversity in CPS molecules could help to evade phage attack. We also examined P24-resistant mutants (B1-4, B1-5, B1-6, and other 20 mutants) isolated from the intestinal tract of treated mice and found that in vivo P24-resistant mutants also had modifications in gene *wcaJ* and *mshA*. In particular, *wcaJ* was interrupted by several different insertion sequences at a few locations in P24-resistant mutants, suggesting that *wcaJ* is a hotspot of mutations to mediate resistance to phages.

In addition, we analyzed the mutants resistant to both P39 and P24 (B2-1, B2-2, and B2-3) and found that another glycosyltransferase-encoding *gene epsJ* also mediated resistance to phage (P39 in this case). EpsJ is a glycosyltransferase involved in the exopolysaccharide synthesis^41^. As expected, the adsorption of P39 to B2-1 and B2-3 was 0%. The receptor for P39 is therefore likely located on the sugar chains on the cell surface. When the parental strain B0 became resistant to P24, extracellular sugar chains could be exposed due to the reduction of outer capsular content, and the P24-resistant mutants, therefore, became susceptible to P39. The adsorption of P39 to B2-2 was similar to those of B1-1 and B1-2. Therefore, the phage resistance mechanism of B2-2 was not due to a change in the receptor-binding site. We found and confirmed a mutation of the cyclic di-GMP phosphodiesterase PdeC in B2-2 leads to phage resistance similar to those reported previously for abortive-infection defense systems^17,18^. All the above findings indicate that bacteria can escape the attack of phage through sophisticated multiple approaches. There is still a long way to overcome phage resistance.

We are aware of the limitations of this study. First, as P24 could not be detected from feces by oral administration, we administered P24 by enema but P39 by oral. The different administration routes of the two phages may introduce a confounding factor to interpret the results. As discussed above, microcapsules may therefore be required to protect phage particles. Second, we only studied colonization due to a single strain. However, for patients infected by multiple strains of CRKP, the phage combination against a single strain may not work and more phages in combination may be needed. Third, although P24 was able to lyse all tested ST11 KL64 strains in spot assays, it formed a turbid zone rather than a clear one to indicate lysis against ST11 KL64 strain 090527 in the EOP assay. This suggests that P24 could bind to this strain but did not produce sufficient progeny phages to form plaques^42^. The exact underlying mechanism for this discrepancy warrants further explorations.

## Methods

### Bacteria and antimicrobial susceptibility

The KPC-2-producing CRKP clinical strain 015134 (assigned B0 here for brevity) was used for phage isolation. Strain B0 belongs to the capsule type KL64 and sequence type (ST) 11, the dominant CRKP type in China^19,20^. Additional CRKP strains (*n* = 20) of different clonal background defined by STs, capsule types, and clones based on whole-genome sequencing and analysis were used to examine the lysis spectrum of the phages (Supplementary Table 1). The number of single nucleotide polymorphisms (SNPs) between ST11 CRKP strains to demonstrate their different clonalities are shown in Supplementary Table 11. Minimum inhibitory concentrations (MICs) of 13 antimicrobial agents were determined using the broth microdilution method according to the Clinical and Laboratory Standards Institute (CLSI) guidelines^43^. *Pseudomonas aeruginosa* ATCC 27853 and *Escherichia coli* ATCC 25922 were used as a control for in vitro susceptibility testing.

### Phage isolation

The phages described in this study were isolated from sewage samples collected at the untreated influx of the wastewater processing station at West China Hospital in 2019. We performed two rounds of phage isolation. In the first round, we used strain B0 as the indicator host to isolate phages lytic against this strain using the double-layer agar method^44^. Sewage samples were centrifuged at 12,000 × *g* for 10 min to remove the precipitated impurities and filtered through a 0.22 µm filter (Labgic Technology; Beijing, China) to remove bacterial debris. We added 1 mL of B0 culture at the logarithmic phase and 2 mL of 10 × LB broth (Hopebio; Qingdao, China) into 17 mL filtered sewage supernatants in a 50 mL tube. The co-culture was incubated at 37 °C for 4 h and thereafter centrifuged at 12,000 × *g* at 4 °C for 2 min. Supernatants were passed through a 0.22 μm filter (Labgic Technology) and were then diluted in ten-fold concentration gradients using Tris-HCl-MgSO_4_ buffers. Filtered supernatant (10 µL) of different dilutions was mixed with 100 µL B0 culture at logarithmic phase and 5 mL 0.7% semi-solid LB agar (Hopebio) and were then poured onto a 1.5% LB agar plate. After overnight incubation at 37 °C, the formation of plaques could be observed. Isolated plaques were picked by micropipette aspiration using filter tips and soaked in Tris-HCl-MgSO_4_ buffer and were further purified at least 3 times until the formation of uniform plaques.

Using the above procedure, we obtained a lytic phage named 066024 (assigned P24 for brevity). We used the double-layer agar method^44^ to examine whether P24-resistant mutants could emerge. Briefly, we combined 10 µL high-titer phage stocks at 10^11^ plaque-forming units (PFU)/mL with 100 µL B0 culture at logarithmic phase and 5 mL of 0.7% semi-solid LB agar, gently vortexed, then poured onto 1.5% LB agar plates. After overnight incubation at 37 °C, individual colonies that grew on the plates were phage P24-resistant mutants and three individual colonies, assigned B1-1, B1-2, and B1-3, respectively, were randomly selected for further studies. P24 resistance of the three mutants was confirmed using the spot assay^13^. Briefly, each of the three mutant strains was streaked on a LB agar plate and 2 μL high-titer phage stocks at 10^11^ PFU/mL were dropped onto the agar overlaid by the bacterial strain. After overnight incubation at 37 °C, the absence of a plaque was regarded as phage resistance.

In the second round, we used a P24-resistant mutant, B1-1, as the indicator host strain to isolate lytic phages against this mutant as described above. We, therefore, obtained the second phage, named 066039 (assigned P39 here for brevity). We also isolated three P39-resistant mutants using the same procedure as described above, which are assigned B2-1, B2-2, and B2-3, respectively.

### Phage host range determination

As the phage host range is an essential factor for phage therapy and decolonization, we performed spot assays and efficiency of plating (EOP) experiments to determine the host range of phages P24 and P39 against the additional 20 CRKP strains of different STs or clones (Supplementary Table 1) using a serial dilution (10^2^–10^10^ PFU/mL)^42^. The presence of a clear zone and lysis plaque was recorded as the strain being susceptible to the tested phage. EOP value was the titer of the phage (P24 or P39) on its susceptible bacterial lawn compared to that on the host strain (015134 for P24 and B1-1 for P39).

### pH and temperature adaptation

To test the stability of phages in different pH and temperatures, Tris-HCl-MgSO_4_ buffers with different pH values (2–13) were prepared. A 100 μL aliquot of 10^8^ PFU/mL phages was mixed with 900 μL Tris-HCl-MgSO_4_ buffer at each pH value. After incubation at 25 °C for 1 h, the phage titer was determined using the double-layer agar method^44^. Thermotolerance of phages was tested at 4, 25, 37, 50, 60, and 70 °C for 1 h in a water bath. A 100 μL aliquot at each temperature was retrieved to determine the phage titer using the double-layer agar method^44^. The above experiments were performed in triplicate.

### Multiplicity of infection (MOI) assay

MOI is the ratio of infecting phages to susceptible hosts. Under the optimal MOI, phage lyses the same number of bacteria to produce the highest amount of progeny phages. We, therefore, explored various MOI to determine the optimal one. Phages were adjusted to the density of 10^8^, 10^7^, 10^6^, 10^5^, and 10^4^ PFU/mL, respectively. The phage and host bacterial strain (P24 and strain B0, P39, and strain B1-1) were mixed with MOIs of 100, 10, 1, 0.1, and 0.01, respectively. After incubation for 3 h at 37 °C, the phage titers were measured by the double-layer agar method^44^ as described above to determine the highest number of phages as the optimal MOI.

### Phage adsorption and one-step growth

To understand the ability of the phage to adsorb to the host strain, we mixed 10 mL bacterial host strain (B0 for P24 and B1-1 for P39) at a concentration of 10^8^ colony forming units (CFU)/mL with 100 μL phage of 10^8^ PFU/mL at a 0.01 MOI, the optimal MOI. At 0, 3, 6, 9, 12, 15, and 20 min, 1 mL co-cultures were retrieved to count phage titers. To explore whether phage resistance is due to a decrease in phage adsorption, we determined adsorption of P24 to the three P24-resistant mutants (B1-1, B1-2, and B1-3) and that of P39 to the three P39-resistant mutants (B2-1, B2-2, and B2-3). To estimate the latent period and the burst size of P24 and P39, we performed one-step growth experiments^45^. Each phage (P24 or P39) was added at a 0.01 MOI as described above and was allowed to be adsorbed for 5 min at 37 °C. One mL of the mixture was retrieved and centrifuged at 12,000 × *g* for 1 min to remove the free, unabsorbed phage particle and the pellets containing infected cells were re-suspended in 1 mL LB broth. The re-suspended mixture was further diluted in tubes A, B, and C by 100, 1000, and 10,000 times, followed by incubation at 37 °C. Samples were retrieved from the appropriate tube (A, B, or C) at various time points up to 110 min to determine phage titers as described above. Phage-infected cells were calculated by subtracting the number of free phages in the filtered aliquot (through 0.22 μm membrane) from that of the un-filtered counterpart at the beginning. Burst size was defined as the value obtained by dividing the average phage-infected cells into the stationary phase progeny counts. The above experiments were performed in triplicate.

### Transmission electron microscopy

We performed transmission electron microscopy (TEM) imaging to observe the morphology of phages. Phage particles were prepared according to conventional methodology^44^. The phage particles were dropped on carbon film-coated copper grids (Carbon Type-B 100 mesh; Zhongjingkeyi Technology; Beijing, China) for 10 min and then a drop of 2.0% (w/v) uranyl acetate was added for negative staining. The morphology and the size of the phage were visualized using a JEM-1400PLUS transmission electron microscope (JEOL; Tokyo, Japan) at an accelerating voltage of 80 kV.

### In vitro phage bacteriolytic assay

The bacteriolytic activity of P24 alone or in combination with P39 against strain B0 and that of P39 against mutant B1-1 were tested. Phage suspensions were added to a culture of bacterial strain at pre-logarithmic phase (OD_600_ = 0.20, around 10^8^ CFU/mL) at a 0.01 MOI. Aliquots (1.5 mL) of the co-culture were retrieved at 1-h intervals for up to 8 h to measure changes in bacteria density using a spectrophotometer (Youke; Shanghai, China). Bacterial strains cultured in the absence of phages and LB broth without bacteria were used as positive and negative controls, respectively. The assays were performed in triplicate.

### Phage minimum inhibitory titer test

To determine the minimum inhibitory titer of phages P24 and P39 against their target bacterial strains, a solution of each phage was diluted to 10^9^, 10^8^, 10^7^, 10^6^, 10^5^, and 10^4^ PFU/mL in Mueller–Hinton broth (Hopebio). Aliquots (100 μL) of each dilution were pipetted into a 96-well plate containing 100 μL 10^6^ CFU/mL strain B0 (for P24) or B1-1 (for P39) in each well to obtain a 1,000 to 0.01 MOI. We also determined the minimum inhibitory titer of the combination of P24 and P39 against strain B0. The phage-bacteria co-culture was incubated at 37 °C, and bacterial densities were measured using a spectrophotometer (Youke) at 24 h and 48 h. Culture of the corresponding bacterial strain in the absence of phages and Mueller–Hinton broth without bacteria nor phages were used as positive and negative controls, respectively. The experiments were performed in triplicate.

### Human bactericidal serum assay

In order to detect whether phage-resistant CRKP mutants were more likely to be killed by serum components, we performed the serum bactericidal assay^46^. The parental strain B0, its P24-resistant mutants (B1-1, B1-2, and B1-3), and P39-resistant mutants (B2-1, B2-2, and B2-3) were tested. We mixed 100 μL of each bacterial strain at 10^6^ CFU/mL with 300 μL human serum with informed consent from healthy volunteers. The serum-strain mixture was incubated at 37 °C without shaking, and 100 μL aliquots were retrieved at 0, 1, 2, and 3 h and then streaked on LB agar plates for colony counting.

### *Galleria mellonella* larvae infection model

To detect whether the virulence of phage-resistant mutants was attenuated, we used the *G. mellonella* (wax moth) larvae infection model, which has been widely used in bacterial virulence assay^47,48^. Briefly, 16 larvae of 250–350 mg in weight (Huiyude; Tianjin, China) were randomly selected for each group. Strain B0, P24-resistant mutants (B1-1, B1-2, and B1-3), P39-resistant mutants (B2-1, B2-2, and B2-3), and two transformants (B1-1::p*mshA*, and B1-2::p*wcaJ*, see below for detailed information) were washed using phosphate-buffered saline (PBS; Beyotime, Shanghai, China) and were adjusted to 0.5 McFarland (around 10^8^ CFU/mL). We injected 10 μL bacterial cultures (10^6^ CFU) into each larvae using a microsyringe (Gaoge; Shanghai, China), while larvae injected with PBS alone were used as negative controls. The larvae were then incubated at 37 °C, and the number of dead larvae was recorded at 12-h intervals up to 72 h. Larvae death is defined by the complete absence of movement under repeated agitations.

### Preparation and quantification of capsules

Previous studies^37,38^ have found that capsule changes can lead to phage resistance. To detect whether there were changes in capsule production in phage-resistant mutants, we prepared and quantified levels of capsules. Glucuronic acid content was extracted and quantified using a colorimetric assay^49,50^. Briefly, 500 µL bacterial cultures growing to stationary phase were mixed with 100 µL 1% zwittergent 3-14 detergent (Macklin Biochemical; Shanghai, China) in 100 mM citric acid (pH 2.0). After incubation at 50 °C for 30 min, the mixtures were centrifuged at 10,000 × *g* for 5 min. A 250 µL aliquot of the supernatant was transferred to a new tube, and 1 mL of absolute ethanol was added to precipitate the capsule polysaccharide (CPS) at 4 °C for 8 h. The supernatant was thoroughly and carefully discarded, while the pellets were allowed to dry. Pellets were then dissolved in 200 µL water and were mixed with 1,200 µL of 12.5 mM borax (Macklin) concentrated in H_2_SO_4_. Mixtures were vigorously vortexed, were incubated at 100 °C for 5 min, and then were cooled to room temperature. After 20 µL of 0.15% 3-hydroxydiphenol in 0.5% NaOH solution were added, the absorbance was measured at 490 nm using a spectrophotometer (Youke) and used to establish a standard curve of glucuronic acid, from which the CPS content was determined.

### Whole-genome sequencing

Genomic DNA of phages P24 and P39 was prepared from a high-titer stock of phage particles at 10^11^ PFU/mL using a Phage DNA isolation Kit (Norgen Biotek; Thorold, Canada) following the manufacturer’s protocol. Total genomic DNA of strain B0, its P24-resistant mutants (B1-1, B1-2, and B1-3), three P24-resistant mutants isolated from mouse feces (B1-4, B1-5, and B1-6, see below), and P39-resistant mutants (B2-1, B2-1, and B2-3) were prepared from bacterial cultures at mid-logarithmic phase using a DNeasy Blood & Tissue Kit (QIAGEN; Hilden, Germany). The prepared DNA was ultrasonically sheared into 350 bp prior to the construction of 150-bp paired-end libraries, which were sequenced using HiSeq X10 (Illumina; San Diego, CA, USA). The generated raw reads were trimmed using Trimmomatic v0.38^51^ prior to assembly into draft genomes using SPAdes v3.13.0^52^ under the careful mode.

Antimicrobial resistance genes of bacterial strains were identified using AMRFinderPlus v3.9^53^. Capsule typing and virulence factor detection were performed using Kleborate v2.0.0 (https://github.com/katholt/Kleborate). Genome sequences were annotated using Prokka v1.12^54^, Rapid Annotations Subsystems Technology (RAST, http://rast.nmpdr.org/) and were manually curated using BLASTp (https://blast.ncbi.nlm.nih.gov/Blast.cgi). Ribosome binding sites (RBS) of genes were identified using Prodigal v2.6.3 (https://github.com/hyattpd/Prodigal) and predicted genes without RBS were removed. Phage gene maps were constructed using SnapGene v5.3 (https://www.snapgene.com/). PHACTS^55^ was used to determine the lytic or lysogenic lifestyle of the two phages. To further determine the taxonomic position of P24 and P39, the amino acid sequence of DNA polymerase of P24 and the major capsid protein of P39 was aligned with those of phages belonging to the genus *Przondovirus* and the genus *Webervirus*, respectively, all of which were retrieved from NCBI, using Phylogeny.fr (http://www.phylogeny.fr/index.cgi). Overall DNA sequence homolog was defined as coverage multiplied by identity according to the International Committee on Taxonomy of Viruses (ICTV)^8^. SNPs between the genome sequence of B0 and those of its P24-resistant mutants as well as between that of B1-2 and those of its P39-resistant mutants were identified using Snippy v4.6.0 (https://github.com/tseemann/snippy). Mutations in the CPS synthesis genes *mshA* and *wcaJ*, the exopolysaccharide (EPS) synthesis gene *epsJ*, and the cyclic di-GMP phosphodiesterase-encoding gene *pdeC* were further confirmed using PCR (primers listed in Supplementary Table 12) and Sanger sequencing. Insertion sequences were identified using ISfinder (https://www-is.biotoul.fr/blast.php). The deletion and acquisition of genomic fragments in the phage-resistant mutants were identified using Roary v3.11.2^56^.

### Plasmid stability test

During the study process, we found an IncFIB(K) plasmid carrying a trimethoprim-resistant gene *dfrA12*, assigned pB1-3, lost from strain B1-1 and B1-3, a P24-resistant mutant. To determine whether the loss of pB1-3 was due to exposure to P24, we conducted a plasmid stability test^57^. Briefly, we added 100 μL 10^9^ PFU/mL of phage P24 to 5 mL B0 culture adjusted to 0.5 McFarland (around 10^8^ CFU/mL), while we added 100 μL LB broth to the control group. The co-culture was incubated at 37 °C for 5 h. Ten-fold serial diluted cultures (100 µL) were streaked on chromogenic ager plates (CHROMagar Orientation; CHROMagar; Paris, France) containing 2 µg/mL meropenem (as strain B0 is carbapenem-resistant) and incubated overnight at 37 °C. The presence of *dfrA12* gene representing pB1-3 in colonies was screened by PCR (primers listed in Supplementary Table 12) to determine the frequency of plasmid loss.

### Cloning experiments to investigate phage resistance mechanisms

Cloning experiments were conducted to investigate whether the mutations in the CPS synthesis genes *mshA* and *wcaJ* mediate resistance to phages. The complete sequences of *mshA* and *wcaJ* of strain B0 were obtained by PCR using self-designed primers (Supplementary Table 12). The amplicons were digested using corresponding restriction endonucleases and were then ligated with the cloning vector pBC SK (Stratagene; La Jolla, CA, USA) pretreated with the same endonucleases. The constructed recombinant plasmids were chemically transformed^44^ into P24-resistant mutants B1-1 and B1-2, respectively. The transformants were screened on LB agar plates containing 50 μg/mL chloramphenicol for 24 h at 37 °C. The presence of cloned *mshA* and *wcaJ* gene in transformants was confirmed by PCR with universal primers M13 forward/reverse and subsequent Sanger sequencing. B1-1 transformants containing the *mshA*-carrying recombinant plasmid (assigned B1-1::p*mshA* here) and B1-2 containing the *wcaJ*-carrying recombinant plasmid (B1-2::p*wcaJ*) were tested for the susceptibility to phage P24 and P39 using the spot assay as described above with strains B0 and B1-1 as control.

We also cloned EPS synthesis gene *epsJ* from B1-2 to P39-resistant mutants B2-1 and B2-3 and cloned cyclic di-GMP phosphodiesterase-encoding gene *pdeC* from B1-2 to P39-resistant mutant B2-2, respectively, as described above. The generated transformants B2-1::p*epsJ*, B2-3::p*epsJ*, and B2-2::p*pdeC* which are the corresponding strain containing the *epsJ*- or *pdeC*-carrying recombinant plasmid, were tested for the susceptibility to phage P39 using the spot assay as described above with strain B1-2 used for control.

### Mouse model of CRKP colonization and decolonization

All animal experiments in this study were approved by the Ethics Committee for Laboratory Animals of West China Hospital, Sichuan University, Chengdu, China. Wild-type C57BL/6J mice were obtained from ENSIWEIER (Chengdu, China) and were maintained in a standard animal facility at the Laboratory Animal Center of Sichuan University. Five- to 7-week-old mice (weight, 18–21 g) were used in experiments. To establish the CRKP colonization model, mice (*n* = 22) were given drinking water containing 5 µg/mL meropenem for 3 days to disturb the intestinal flora. Strain B0 was grown to logarithmic phase in LB broth, harvested by centrifugation at 10,000 × *g* for 10 min, washed using PBS, and adjusted to 0.5 McFarland (around 10^8^ CFU/mL). Mice (*n* = 22) were fed with 200 µL 10^5^ CFU/mL of CRKP strain B0 using the feeding needle. To quantify daily bacterial shedding, we gently caressed the abdomen of each mouse and collected one fecal particle with sterilized 1.5 mL centrifuge tubes. Samples were diluted 1:10 (w/v) in PBS and vortexed violently for 5 min. The tubes were stood for 5 min to pellet out larger debris. Ten-fold serial diluted supernatants (100 µL) were streaked on Simmons’ Citrate Agar plates (Hopebio) containing 1% inositol, 2 µg/mL meropenem to select B0 and 4 µg/mL linezolid to inhibit the growth of Gram-positive bacteria from mouse feces. Plates were incubated for 48 h at 37 °C. Shedding of strain B0 was calculated in CFU per gram of feces with a detection limit of 10^2^ CFU/g. Each mouse was given a unique marking and therefore the fecal shedding of each individual mouse could be tracked during the experiment. The stable shedding of B0 from mice for at least 7 days was considered as successful colonization. To verify the absence of any phages able to lyse strain B0 in mice prior to the decolonization experiments, we collected the feces of each mouse for phage isolation using the double-layer agar method^44^ before the administration of phage P24 alone or in combination with P39. For mice (*n* = 8 for each phage administration) treated with P24 alone or in combination with P39, we collected feces the day after phage administration and determined the phage titer using the double-layer agar method^44^. To examine the safety of phages, we continuously fed healthy mice (*n* = 6) with water containing 10^9^ PFU/mL of P39 for 7 days, while P24 was given by enema at 10^9^ PFU/mouse once daily for 3 days. Mice were visually checked for diarrhea, mental depression, weight loss, death, and other adverse reactions once daily.

To determine the decolonization efficacy of phages in mice, P24 dissolved in PBS was administered by enema at 10^9^ PFU/mouse once daily for 3 days after successful colonization of strain B0 in the P24-treated group (*n* = 8). While in the P24 and P39 combination group (*n* = 8), B0-colonized mice were additionally fed with water containing 10^9^ PFU/mL of P39 to the end of this experiment for 7 days. In the control group (*n* = 6), B0-colonized mice were treated with 100 µL PBS buffer once daily for 3 days by enema. The ability of P24 alone or in combination with P39 to decolonize CRKP from the intestinal tract of mice was investigated using colony counting for strain B0 using Simmons’ Citrate Agar plates as described above.

### Detection of *wcaJ* and *mshA* mutations in P24-resistant mutants from mouse feces

Three randomly selected P24-resistant mutants (B1-4, B1-5, and B1-6) isolated from mouse feces were subjected for whole-genome sequencing as described above. Twenty additional P24-resistant mutants were randomly selected from the culture of mouse feces and were examined for mutations in *wcaJ* and *mshA* by PCR (primers listed in Supplementary Table 12) and Sanger sequencing for PCR amplicons.

### Statistics and reproducibility

Statistical analysis was performed using SPSS 22.0 (IBM Analytics; Armonk, NY, USA) to plot survival curves with the Kaplan–Meier method following a log-rank test^58^ for calculating the survival differences in different *G. mellonella* larvae groups. To test the difference of the CPS between B0 and its P24-resistant mutants, we used an unpaired two-sided Student’s *t*-test. GraphPad Prism 8.0 (GraphPad Software, Inc.; San Diego, CA, USA) was used to plot mice bacterial shedding map, and shedding differences were determined using the Mann–Whitney *U* test^59^. A < 0.05 *P*-value was statistically significant.

### Reporting summary

Further information on research design is available in the Nature Research Reporting Summary linked to this article.

## Supplementary information

Supplementary Information

Description of Additional Supplementary Files

Supplementary Data 1

Supplementary Data 2

Supplementary Data 3

Supplementary Data 4

Reporting Summary

## Data Availability

The sequences of phage P24 and P39 have been deposited into GenBank with assigned accession numbers MW042792.1 and MW042802.1, respectively. Draft genomes of *K. pneumoniae* B0, B1-1, B1-2, B1-3, B1-4, B1-5, B1-6, B2-1, B2-2, and B2-3 have been deposited into GenBank under the accession numbers NWCG00000000, JADLJA000000000, JADLIZ000000000, JADLIY000000000, JAHAWF000000000, JAHAWG000000000, JAHAWH000000000, JAHAWI000000000, JAHKSV000000000, and JAHAWJ000000000, respectively. The source results are shown in Supplementary Data 1–4.
