# Supplementary material for: Characterization of phage resistance and phages capable of intestinal decolonization of carbapenem-resistant Klebsiella pneumoniae in mice
Source: Commun Biol. 2022 Jan 13;5:48. doi: 10.1038/s42003-022-03001-y (PMC8758719; doi:10.1038/s42003-022-03001-y)
Supplement: Supplementary file 1 — Supplementary Information [file 42003_2022_3001_MOESM1_ESM.pdf]

Supplementary Figure 1

Characteristics of growth and stability of P24 and P39.

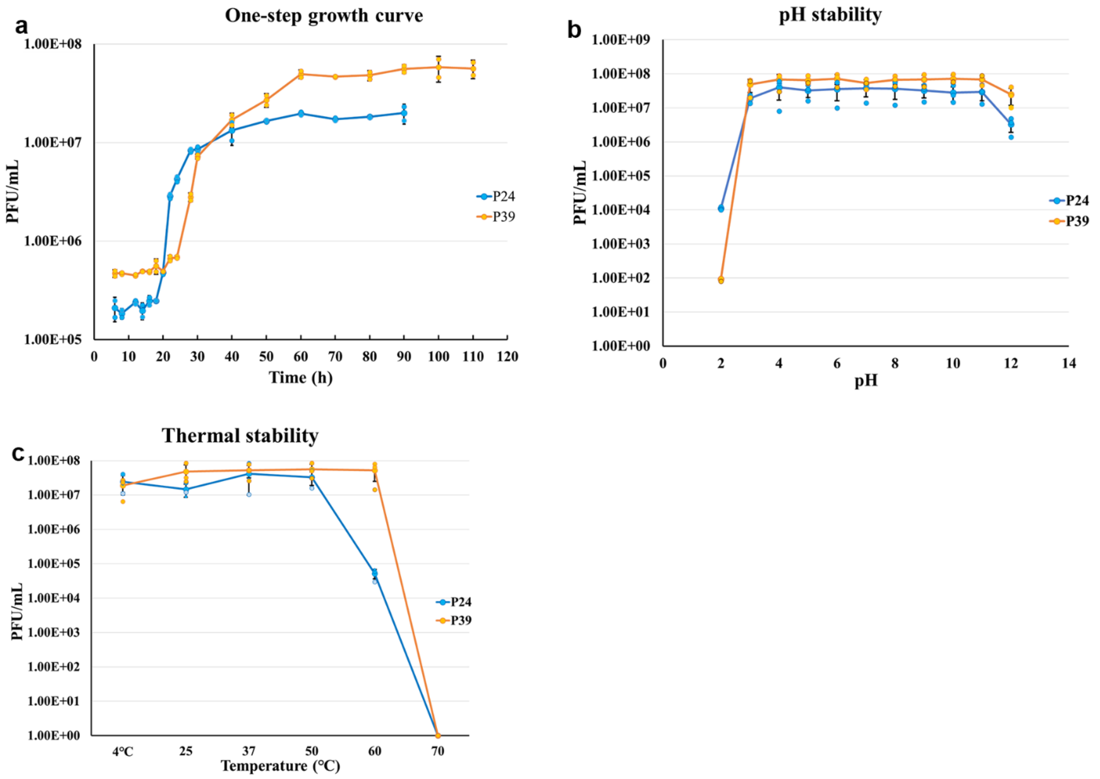

(a) One-step growth curve. The latent period for P24 and P39 was 20 and 25 min, respectively. An average burst size for P24 and P39 was 86 and 107 PFU per infected cell, respectively; (b) Stability of phage under different pH values. Both phages remained relatively stable within a pH range of 3–11 for 1 h. Incubation at pH 2 caused a 3- and 6-log decrease in the phage titer of P24 and P39, respectively; c) Stability at various temperatures. Both phages remained stable at temperature of 4-50 °C for 1 h. The titer of P24 and P39 declined by 3- and 1-log at 60 °C, respectively. Both phages were completely inactivated at 70 °C. Data are shown mean ± SD (n = 3) and error bars represent standard deviation. Individual data were shown in colored points. The source results are shown in Supplementary Data 4.

Supplementary Figure 2

Phylogenetic trees of P24, P39 and their closely-related phages

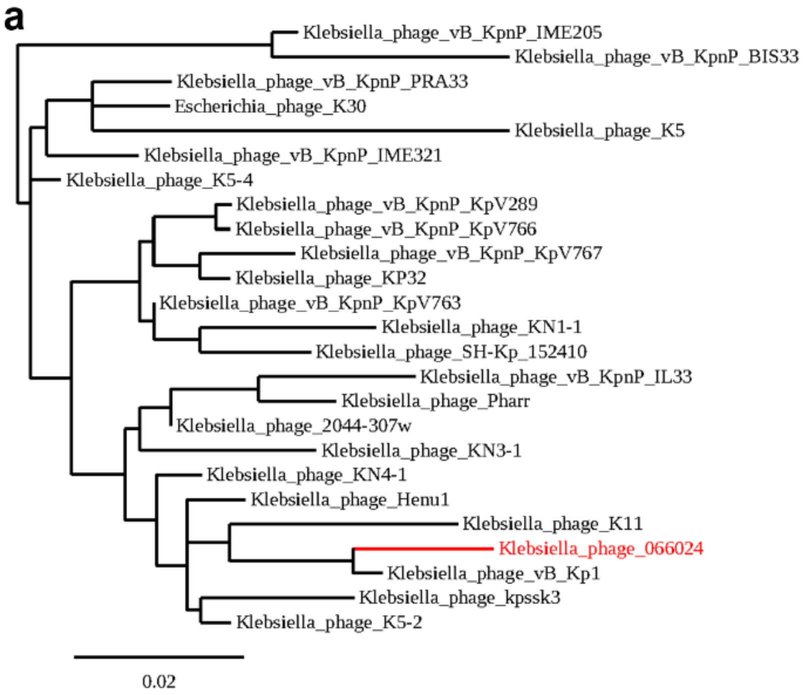

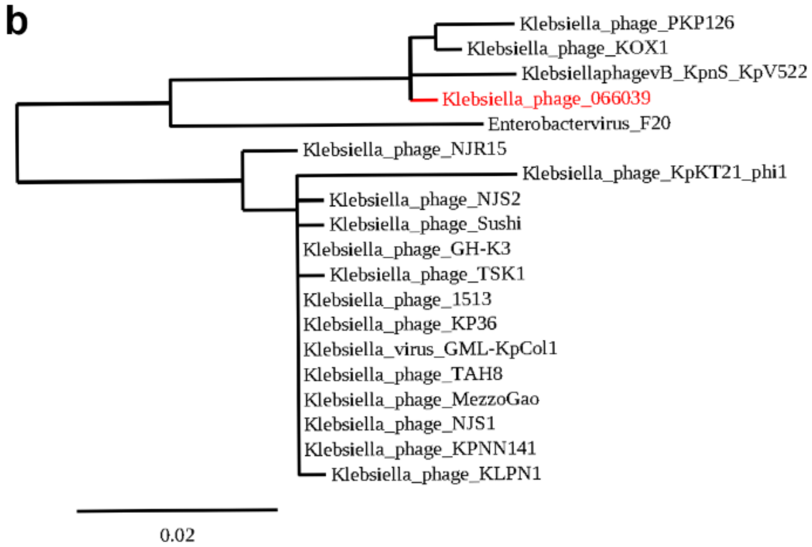

(a) Phylogenetic tree based on the amino acid sequence of the DNA polymerase of P24 (066024) and phages belonging to the genus *Przondovirus* of the *Studiervirinae* subfamily. (b) Phylogenetic tree based on the amino acid sequence of the major capsid protein of P39 (066039) and phages belonging to the *Webervirus* genus of the family *Drexlerviridae*. The trees were inferred using “One Click” at http://www.phylogeny.fr/.

Supplementary Table 1

CRKP strains used in this study and the host range of phages

Strains	Accession No.	ST	Carbapene-mase genes	Capsule type	Source	P24		P39		EOP (%)
						Spot	Plaque	Spot	Plaque	
B0 (015134)	NWCG00000000	11	$\textit{bla}_{\text{KPC-2}}$	KL64	Rectal swab	+	+	-		100
020037	CP036371-CP036375	11	$\textit{bla}_{\text{KPC-2}}$	KL64	Sputum	+	+	-		0.1-0.2
090527	JAEMHO000000000	11	$\textit{bla}_{\text{KPC-2}}$	KL64	Ascites	+	-	-		
015318	PWCG00000000	11	$\textit{bla}_{\text{KPC-2}}$	KL64	Rectal swab	+	+	-		0.7-0.9
015584	QJMQ00000000	11	$\textit{bla}_{\text{KPC-2}}$	KL64	Rectal swab	+	+	-		2.3-3.4
020003	CP031717-CP031721	11	$\textit{bla}_{\text{KPC-2}}$	KL64	Urine	+	+	-		3.5-4.6
015785	JAEMHP000000000	11	$\textit{bla}_{\text{KPC-2}}$	KL64	Rectal swab	+	+	-		115.4-230.8
020030	CP028788-CP028793	11	$\textit{bla}_{\text{KPC-2}}$	KL47	Tissue	-		-		
020143	CP028543-CP028548	11	$\textit{bla}_{\text{KPC-2}}$	KL47	Secretion	-		-		
015712	JAEMHQ000000000	11	$\textit{bla}_{\text{KPC-2}}$	KL47	Rectal swab	-		-		
020058	NWDZ00000000	11	$\textit{bla}_{\text{KPC-2}}$	KL47	Sputum	-		-		
020046	CP028779-CP028783	11	$\textit{bla}_{\text{NDM-5}}$	KL39	Secretion	-		-		
015581	QJMP00000000	11	$\textit{bla}_{\text{KPC-2}}$	KL39	Rectal swab	-		-		
020077	NWMY00000000	37	$\textit{bla}_{\text{NDM-1}}$	KL38	Sputum	-		-		
020070	NGZF00000000	37	$\textit{bla}_{\text{NDM-1}}$	KL38	Sputum	-		-		
020135	CP037963-CP037967	1	$\textit{bla}_{\text{NDM-1}}$	KL45	Sputum	-		-		
020136	NWFF00000000	1	$\textit{bla}_{\text{NDM-1}}$	KL45	Blood	-		-		
020117	PWAI00000000	45	$\textit{bla}_{\text{NDM-1}}$	KL5	Sputum	-		-		
020035	CP045988-CP045992	45	$\textit{bla}_{\text{KPC-2}}$	KL62	Blood	-		-		
020083	NOKM00000000	15	$\textit{bla}_{\text{NDM-5}}$	KL106	Secretion	-		-		
020052	PWAS00000000	54	$\textit{bla}_{\text{IMP-4}}$	KL14	Feces	-		-		

P24-resistant mutants								
B1-1	JADLJA000000000	11	*bla*$_{KPC-2}$	KL64	**-**	**+**	**+**	100
B1-2	JADLIZ000000000	11	*bla*$_{KPC-2}$	KL64	**-**	**+**	**+**	75-100
B1-3	JADLIY000000000	11	*bla*$_{KPC-2}$	KL64	**-**	**+**	**+**	66.7-133.3
P39-resistant mutants								
B2-1	JAHAWI000000000	11	*bla*$_{KPC-2}$	KL64	-	-		
B2-2	JAHKSV000000000	11	*bla*$_{KPC-2}$	KL64	-	-		
B2-3	JAHAWJ000000000	11	*bla*$_{KPC-2}$	KL64	-	-		

+, phage formed a clear zone or plaque; −, phage form no clear zone nor plaque.

Supplementary Table 2

Genome information of the two phages in this study

	P24	P39
Genome size	40,770 bp	51,633 bp
tRNA	0	0
GC%	53.04%	51.46%
Total Proteins	50	81
Proteins of known function	30	40
Accession No.	MW042792.1	MW042802.1
Classification	*Przondovirus* genus	*Webervirus* genus

Supplementary Table 3

Annotation of the sequence of phage P24 (also called 066024, accession no. MW042792.1).

contig_id	feature_id	start	stop	strand	rbs_motif	rbs_spacer	function
066024	fig|066024.3.peg.1	16	969	+	AGGAG	5-10bp	Phage capsid and scaffold
066024	fig|066024.3.peg.2	1111	2142	+	AGGAG	5-10bp	Phage capsid and scaffold
066024	fig|066024.3.peg.3	2199	2420	+	GGAG/GAGG	5-10bp	hypothetical protein
066024	fig|066024.3.peg.4	2485	3063	+	AGGAG	5-10bp	Phage tail fiber protein / T7-like tail tubular protein A
066024	fig|066024.3.peg.5	3086	5461	+	AGGAG	5-10bp	Phage tail fiber protein / T7-like tail tubular protein B
066024	fig|066024.3.peg.6	5534	5944	+	GGA/GAG/AGG	5-10bp	Phage internal (core) protein
066024	fig|066024.3.peg.7	5947	6537	+	AGGAG	5-10bp	Phage internal (core) protein
066024	fig|066024.3.peg.8	6537	8792	+	AGGAGG	5-10bp	Phage internal (core) protein
066024	fig|066024.3.peg.9	8809	9216	+	AGGAG	5-10bp	hypothetical protein
066024	fig|066024.3.peg.10	9200	13168	+	GGAGG	3-4bp	Phage internal (core) protein
066024	fig|066024.3.peg.11	13230	16283	+	AGGAGG	5-10bp	Phage tail fibers*
066024	fig|066024.3.peg.12	16294	16503	+	AGGAGG	5-10bp	Phage holin, class II
066024	fig|066024.3.peg.13	16537	16794	+	AGGAG	5-10bp	DNA packaging protein A, T7-like gp18
066024	fig|066024.3.peg.14	16891	17337	+	GGAGG	3-4bp	Phage endopeptidase (EC 3.4.-.-) Rz
066024	fig|066024.3.peg.15	17334	19091	+	GGAGG	5-10bp	Phage DNA packaging
066024	fig|066024.3.peg.16	19336	19485	+	AGGAGG	3-4bp	hypothetical protein
066024	fig|066024.3.peg.17	20650	20829	+	GGAG/GAGG	5-10bp	hypothetical protein
066024	fig|066024.3.peg.18	20896	21348	+	AGGA	5-10bp	S-adenosyl-L-methionine hydrolase, phage-associated
066024	fig|066024.3.peg.19	21348	21494	+	GGAGG	5-10bp	hypothetical protein
066024	fig|066024.3.peg.20	21472	21669	+	GGA/GAG/AGG	5-10bp	hypothetical protein
066024	fig|066024.3.peg.21	21653	21832	+	GGA/GAG/AGG	5-10bp	hypothetical protein
066024	fig|066024.3.peg.22	21868	22884	+	AGGA	5-10bp	Phage protein kinase (EC 2.7.11.1)
066024	fig|066024.3.peg.23	22954	25674	+	GGAG/GAGG	5-10bp	DNA-directed RNA polymerase (EC 2.7.7.6)
066024	fig|066024.3.peg.24	25773	26333	+	GGAG/GAGG	5-10bp	hypothetical protein
066024	fig|066024.3.peg.25	26423	26599	+	AGGA	5-10bp	hypothetical protein
066024	fig|066024.3.peg.26	26603	26860	+	AGGA	5-10bp	hypothetical protein
066024	fig|066024.3.peg.27	26961	28010	+	GGAG/GAGG	5-10bp	DNA ligase, phage-associated
066024	fig|066024.3.peg.28	28210	28473	+	AGGAG	5-10bp	hypothetical protein
066024	fig|066024.3.peg.29	28466	28912	+	AGGAGG	5-10bp	hypothetical protein
066024	fig|066024.3.peg.30	28899	29024	+	GGA/GAG/AGG	5-10bp	hypothetical protein

066024	fig|066024.3.peg.31	28990	29154	+	AGGAG	5-10bp	Host RNA polymerase inhibitor, T7-like gp2
066024	fig|066024.3.peg.32	29220	29402	+	AGGAGG	5-10bp	hypothetical protein
066024	fig|066024.3.peg.33	29415	30113	+	AGGAG	5-10bp	T7-like phage ssDNA-binding protein
066024	fig|066024.3.peg.34	30113	30562	+	3Base/5BMM	13-15bp	T7-like phage endonuclease (EC 3.1.21.2)
066024	fig|066024.3.peg.35	30565	31020	+	AGGAG	5-10bp	Phage lysin, N-acetylmuramoyl-L-alanine amidase (EC 3.5.1.28)
066024	fig|066024.3.peg.36	31197	31316	+	GGAG/GAGG	5-10bp	hypothetical protein
066024	fig|066024.3.peg.37	31517	33043	+	AGGAGG	5-10bp	T7-like phage primase/helicase protein
066024	fig|066024.3.peg.38	33137	33346	+	AGGAG	5-10bp	hypothetical protein
066024	fig|066024.3.peg.39	33346	33657	+	AGGAG	5-10bp	Tail tubular A-like protein
066024	fig|066024.3.peg.40	33731	34108	+	GGAG/GAGG	5-10bp	hypothetical protein
066024	fig|066024.3.peg.41	34134	36257	+	AGGAG	5-10bp	T7-like phage DNA Polymerase (EC 2.7.7.7)
066024	fig|066024.3.peg.42	36276	36563	+	AGGAG	5-10bp	Phage HNS binding protein
066024	fig|066024.3.peg.43	36560	36769	+	GGAG/GAGG	5-10bp	Phage HNS binding protein
066024	fig|066024.3.peg.44	36766	37149	+	GGAGG	5-10bp	hypothetical protein
066024	fig|066024.3.peg.45	37142	38047	+	GGAGG	5-10bp	T7-like phage exonuclease (EC 3.1.11.3)
066024	fig|066024.3.peg.46	38029	38139	+	GGAG/GAGG	5-10bp	hypothetical protein
066024	fig|066024.3.peg.47	38225	38470	+	AGGAG	5-10bp	DUF2717 domain-containing protein
066024	fig|066024.3.peg.48	38473	38694	+	AGGAG	5-10bp	DUF5476 domain-containing protein
066024	fig|066024.3.peg.49	38696	38956	+	AGGAGG	5-10bp	Tail assembly protein
066024	fig|066024.3.peg.50	38980	40686	+	GGAG/GAGG	5-10bp	Phage collar / T7-like phage head-to-tail joining protein

Genome sequences were annoted using Prokka v1.12 and Rapid Annotations Subsystems Technology (RAST, http://rast.nmpdr.org/), and were manually curated using BLASTp.

*fig|066024.3.peg.11 encodes phage tail fibers, which is also a depolymerase.

Supplementary Table 4
Genome characterization of *Przondovirus* genus phages

Przondovirus genus phage	Accession no.	Size (Kb)	GC%	Protein	tRNAs
Klebsiella phage KP32	GQ413937.1	41.1	52.4	44	0
Klebsiella phage vB_KpnP_KpV767	NC_047772.1	40.4	52.3	52	0
Klebsiella phage vB_KpnP_PRA33	NC_047780.1	40.61	52.5	52	0
Klebsiella virus KP32i192	NC_047968.1	40.64	52.9	41	0
Klebsiella phage Henu1	NC_048138.1	40.35	53.1	42	0
Klebsiella phage vB_KpnP_KpV766	NC_047773.1	41.28	52.6	50	0
Klebsiella phage vB_KpnP_IME321	NC_048014.1	39.91	52.8	49	0
Klebsiella phage vB_KpnP_KpV289	NC_028977.1	41.05	52.6	51	0
Klebsiella virus KP32i194	NC_047969.1	41.16	52.9	41	0
Klebsiella virus KP32i196	NC_047971.1	40.34	52.9	41	0
Klebsiella phage 2044-307w	NC_047842.1	40.05	52.9	44	0
Klebsiella phage K5-4	NC_047799.1	40.16	53.1	42	0
Klebsiella phage vB_Kp1	KT367885.1	40.11	53.3	47	0
Klebsiella phage K11	EU734173.1	41.18	53.2	51	0
Klebsiella phage vB_KpnP_BIS33	NC_047781.1	41.7	52.7	56	0
Klebsiella phage Pharr	NC_048175.1	40.6	53.3	47	0
Klebsiella phage vB_KpnP_KpV763	NC_047771.1	40.77	53.2	49	0
Klebsiella phage K5	KR149291.1	41.7	52.5	46	0
Escherichia phage K30	HM480846.1	40.94	51.4	49	0
Klebsiella phage K5-2	NC_047798.1	41.12	53.3	42	0
Klebsiella phage KN3-1	NC_048131.1	41.06	53.5	24	0
Klebsiella phage vB_KpnP_IL33	NC_047782.1	41.33	52.5	54	0
Klebsiella virus KP32i195	NC_047970.1	40.54	52.9	41	0
**Klebsiella phage 066024**	MW042792.1	40.77	53.2	50	0
Klebsiella phage KN1-1	NC_048129.1	40.24	52.8	22	0
Klebsiella phage kpssk3	NC_048114.1	40.54	52.8	42	0
Klebsiella phage KN4-1	NC_048130.1	41.22	52.9	20	0
Klebsiella phage SH-Kp 152410	NC_047908.1	40.95	52.3	47	0
Klebsiella phage vB_KpnP_IME205	NC_047761.1	41.31	52.2	49	0

Supplementary Table 5
Genome Comparation between *Przondovirus* genus phages and phage P24 (066024) using blastn

Description	Name	Coverage, %	Identity, %	Overall DNA sequence homolog (coverage × identity, %)	Accession
**Klebsiella phage 066024, complete genome**	Klebsiella phage 066024	100	100	100	MW042792.1
Klebsiella phage vB_Kp1, complete genome	Klebsiella phage vB_Kp1	96	95.73	91.9	KT367885.1
Klebsiella virus KP32 isolate 195, complete genome	Klebsiella virus KP32	94	93.54	87.93	NC_047970.1
Klebsiella phage kpssk3, complete genome	Klebsiella phage kpssk3	93	93.62	87.07	NC_048114.1
Klebsiella phage SH-Kp 152410, complete genome	Klebsiella phage SH-Kp 152410	88	94.48	83.14	NC_047908.1
Klebsiella phage K11, complete genome	Klebsiella phage K11	88	93.29	82.1	EU734173.1
Klebsiella phage K5-4, complete genome	Klebsiella phage K5-4	88	92.63	81.51	NC_047799.1
Klebsiella phage Henu1, complete genome	Klebsiella phage Henu1	87	93.41	81.27	NC_048138.1
Klebsiella virus KP32 isolate 192, complete genome	Klebsiella virus KP32	87	92.88	80.81	NC_047968.1
Klebsiella phage KN4-1 DNA, complete genome	Klebsiella phage KN4-1	88	91.7	80.7	NC_048130.1
Klebsiella phage vB_KpnP_KpV767, complete genome	Klebsiella phage vB_KpnP_KpV767	87	92.19	80.21	NC_047772.1
Klebsiella phage 2044-307w, complete genome	Klebsiella phage 2044-307w	87	91.84	79.9	NC_047842.1
Klebsiella phage vB_KpnP_KpV763, complete genome	Klebsiella phage vB_KpnP_KpV763	87	91.79	79.86	NC_047771.1
Klebsiella phage K5-2, complete genome	Klebsiella phage K5-2	87	91.21	79.35	NC_047798.1
Klebsiella phage vB_KpnP_IME321, complete genome	Klebsiella phage vB_KpnP_IME321	87	91.14	79.29	NC_048014.1
Klebsiella phage KP32, complete genome	Klebsiella phage KP32	86	92.1	79.21	GQ413937.1
Klebsiella virus KP32 isolate 194, complete genome	Klebsiella virus KP32	86	92.06	79.17	NC_047969.1
Klebsiella phage vB_KpnP_PRA33, complete genome	Klebsiella phage vB_KpnP_PRA33	87	90.80	79	NC_047780.1
Enterobacteria phage K30, complete genome	Escherichia phage K30	86	91.62	78.79	HM480846.1
Klebsiella phage KN3-1 DNA, complete genome	Klebsiella phage KN3-1	86	91.13	78.37	NC_048131.1
Klebsiella phage vB_KpnP_IME205, complete genome	Klebsiella phage vB_KpnP_IME205	84	93.21	78.3	NC_047761.1
Klebsiella phage vB_KpnP_BIS33, complete genome	Klebsiella phage vB_KpnP_BIS33	86	91.03	78.29	NC_047781.1
Klebsiella phage Pharr, complete genome	Klebsiella phage Pharr	86	90.68	77.98	NC_048175.1
Klebsiella phage vB_KpnP_KpV289, complete genome	Klebsiella phage vB_KpnP_KpV289	87	89.43	77.8	NC_028977.1
Klebsiella phage K5, complete genome	Klebsiella phage K5	86	89.87	77.29	KR149291.1
Klebsiella virus KP32 isolate 196, complete genome	Klebsiella virus KP32	84	91.56	76.91	NC_047971.1
Klebsiella phage KN1-1 DNA, complete genome	Klebsiella phage KN1-1	85	89.93	76.44	NC_048129.1
Klebsiella phage vB_KpnP_KpV766, complete genome	Klebsiella phage vB_KpnP_KpV766	85	89.6	76.16	NC_047773.1
Klebsiella phage vB_KpnP_IL33, complete genome	Klebsiella phage vB_KpnP_IL33	85	87.05	73.99	NC_047782.1

Supplementary Table 6
Annotation of the sequence of phage P39 (accession no. MW042802.1).

contig_id	feature_id	start	stop	strand	rbs_motif	rbs_spacer	function
066039	fig|066039.3.peg.1	636	34	-	AGGA	5-10bp	Phage tail assembly protein
066039	fig|066039.3.peg.2	1348	611	-	GGA/GAG/AGG	5-10bp	Phage tail assembly protein
066039	fig|066039.3.peg.3	2102	1350	-	AGGAG	5-10bp	Phage minor tail protein
066039	fig|066039.3.peg.4	2516	2172	-	GGAG/GAGG	5-10bp	Phage minor tail protein #tail protein M
066039	fig|066039.3.peg.5	5485	2519	-	GGxGG	5-10bp	Phage tail length tape-measure protein
066039	fig|066039.3.peg.6	6161	5835	-	AGGAG	5-10bp	Tail assembly chaperone
066039	fig|066039.3.peg.7	6895	6248	-	AGGAG	5-10bp	Phage tail protein, putative tail protein depolymerase
066039	fig|066039.3.peg.8	7642	6947	-	GGAGG	5-10bp	Phage tail protein, putative tail depolymerase
066039	fig|066039.3.peg.9	8060	7629	-	GGAG/GAGG	5-10bp	DUF4128 domain-containing protein
066039	fig|066039.3.peg.10	8487	8050	-	GGA/GAG/AGG	5-10bp	Putative neck protein
066039	fig|066039.3.peg.11	8857	8480	-	GGAGG	5-10bp	Head-to-tail connector protein
066039	fig|066039.3.peg.12	9279	8863	-	GGAG/GAGG	5-10bp	DUF4054 domain-containing protein
066039	fig|066039.3.peg.13	9639	9331	-	AGGAG	5-10bp	hypothetical protein
066039	fig|066039.3.peg.14	10672	9731	-	AGGAG	5-10bp	Major capsid protein
066039	fig|066039.3.peg.15	11295	10783	-	AGGAG	5-10bp	Capsid decoration protein
066039	fig|066039.3.peg.16	12477	11347	-	GGAG/GAGG	5-10bp	Major capsid protein
066039	fig|066039.3.peg.17	13244	12474	-	AGGAG	5-10bp	Head morphogenesis protein
066039	fig|066039.3.peg.18	14553	13234	-	GGA/GAG/AGG	5-10bp	Phage portal protein
066039	fig|066039.3.peg.19	16204	14600	-	GGA/GAG/AGG	5-10bp	Phage terminase, large subunit
066039	fig|066039.3.peg.20	16738	16214	-	AGGAG	5-10bp	Phage terminase, small subunit
066039	fig|066039.3.peg.21	17079	16816	-	GGA/GAG/AGG	5-10bp	hypothetical protein
066039	fig|066039.3.peg.22	17264	17073	-	GGA/GAG/AGG	5-10bp	hypothetical protein
066039	fig|066039.3.peg.23	17582	17403	-	GGAG/GAGG	5-10bp	hypothetical protein
066039	fig|066039.3.peg.24	17815	17582	-	GGAG/GAGG	5-10bp	hypothetical protein
066039	fig|066039.3.peg.25	18040	17816	-	GGA/GAG/AGG	5-10bp	hypothetical protein
066039	fig|066039.3.peg.26	18685	18110	-	GGA/GAG/AGG	5-10bp	Phage EaA protein
066039	fig|066039.3.peg.27	18954	18682	-	AGGAG	5-10bp	hypothetical protein
066039	fig|066039.3.peg.28	19681	19031	-	GGAG/GAGG	5-10bp	hypothetical protein
066039	fig|066039.3.peg.29	20187	19753	-	AGGAG	5-10bp	hypothetical protein
066039	fig|066039.3.peg.30	20396	20187	-	AGGAG	5-10bp	hypothetical protein
066039	fig|066039.3.peg.31	20656	20477	-	GGAG/GAGG	5-10bp	hypothetical protein
066039	fig|066039.3.peg.32	20898	20653	-	GGA/GAG/AGG	5-10bp	hypothetical protein
066039	fig|066039.3.peg.33	21278	20910	-	GGAG/GAGG	5-10bp	hypothetical protein
066039	fig|066039.3.peg.34	21664	21347	-	GGA/GAG/AGG	5-10bp	hypothetical protein
066039	fig|066039.3.peg.35	21876	21664	-	GGAG/GAGG	5-10bp	hypothetical protein
066039	fig|066039.3.peg.36	22156	21947	-	GGAG/GAGG	5-10bp	hypothetical protein
066039	fig|066039.3.peg.37	22343	22146	-	GGAG/GAGG	5-10bp	hypothetical protein
066039	fig|066039.3.peg.38	22558	22340	-	GGA/GAG/AGG	11-12bp	hypothetical protein
066039	fig|066039.3.peg.39	22809	22555	-	GGA/GAG/AGG	5-10bp	hypothetical protein
066039	fig|066039.3.peg.40	23034	22822	-	AGGAG	5-10bp	hypothetical protein
066039	fig|066039.3.peg.41	23317	23102	-	AGGAG	5-10bp	hypothetical protein
066039	fig|066039.3.peg.42	23541	23320	-	GGA/GAG/AGG	5-10bp	hypothetical protein
066039	fig|066039.3.peg.43	23774	23538	-	GGA/GAG/AGG	5-10bp	hypothetical protein
066039	fig|066039.3.peg.44	24150	23845	-	GGA/GAG/AGG	5-10bp	hypothetical protein
066039	fig|066039.3.peg.45	24677	24147	-	GGA/GAG/AGG	5-10bp	hypothetical protein
066039	fig|066039.3.peg.46	25332	25607	+	AGGAG	5-10bp	hypothetical protein
066039	fig|066039.3.peg.47	25610	25891	+	GGAG/GAGG	5-10bp	hypothetical protein

066039	fig|066039.3.peg.48	25872	26201	+	AGGAG	5-10bp	hypothetical protein
066039	fig|066039.3.peg.49	26228	26566	+	GGAG/GAGG	5-10bp	hypothetical protein
066039	fig|066039.3.peg.50	26639	26833	+	AGGAG	5-10bp	hypothetical protein
066039	fig|066039.3.peg.51	27070	27447	+	AGGA/GGAG/GAGG	11-12bp	hypothetical protein
066039	fig|066039.3.peg.52	27520	27747	+	AGGAG	5-10bp	hypothetical protein
066039	fig|066039.3.peg.53	27747	28088	+	GGAG/GAGG	5-10bp	hypothetical protein
066039	fig|066039.3.peg.54	28081	28320	+	GGAGG	5-10bp	Putative cytosine DNA methylase
066039	fig|066039.3.peg.55	28328	29020	+	GGAG/GAGG	5-10bp	DNA cytosine methyltransferase
066039	fig|066039.3.peg.56	29093	29302	+	GGAG/GAGG	5-10bp	hypothetical protein
066039	fig|066039.3.peg.57	29292	29474	+	GGxGG	5-10bp	hypothetical protein
066039	fig|066039.3.peg.58	29476	29913	+	AGGA	5-10bp	hypothetical protein
066039	fig|066039.3.peg.59	30038	31606	+	AGGAG	5-10bp	Putative helicase
066039	fig|066039.3.peg.60	31610	32071	+	GGAG/GAGG	5-10bp	hypothetical protein
066039	fig|066039.3.peg.61	32565	32140	-	AGGA	5-10bp	Putative spanin
066039	fig|066039.3.peg.62	33044	32562	-	AGGAG	5-10bp	Phage lysin/Phage lysozyme
066039	fig|066039.3.peg.63	33261	33046	-	GGAG/GAGG	5-10bp	Phage holin
066039	fig|066039.3.peg.64	33968	33396	-	GGAG/GAGG	5-10bp	Putative nucleoside triphosphate hydrolase
066039	fig|066039.3.peg.65	34459	33965	-	AGGA	5-10bp	3'-phosphatase, 5'-polynucleotide kinase
066039	fig|066039.3.peg.66	35627	34497	-	GGA/GAG/AGG	5-10bp	hypothetical protein
066039	fig|066039.3.peg.67	35971	35723	-	AGGAG	5-10bp	hypothetical protein
066039	fig|066039.3.peg.68	36207	35971	-	AGGAG	5-10bp	hypothetical protein
066039	fig|066039.3.peg.69	36508	36272	-	GGA/GAG/AGG	5-10bp	hypothetical protein
066039	fig|066039.3.peg.70	37243	36512	-	AGGA	5-10bp	DNA adenine methyltransferase
066039	fig|066039.3.peg.71	37472	37245	-	GGAG/GAGG	5-10bp	PDDEXK-like family protein
066039	fig|066039.3.peg.72	37952	37545	-	GGAGG	5-10bp	VRR-NUC domain-containing protein
066039	fig|066039.3.peg.73	39985	37949	-	AGGA	5-10bp	DNA helicase, phage-associated
066039	fig|066039.3.peg.74	40076	40477	+	GGAG/GAGG	5-10bp	Transcriptional regulator
066039	fig|066039.3.peg.75	40553	41515	+	GGAGG	5-10bp	DNA primase/helicase
066039	fig|066039.3.peg.76	42012	43058	+	AGGAG	5-10bp	Phage exonuclease
066039	fig|066039.3.peg.77	43118	43774	+	AGGA	5-10bp	Phage-associated recombinase
066039	fig|066039.3.peg.78	43811	44275	+	AGGA	5-10bp	Putative ssDNA binding protein
066039	fig|066039.3.peg.79	44368	47046	+	GGAG/GAGG	5-10bp	Phage tail fibers
066039	fig|066039.3.peg.80	48030	47074	-	GGAGG	5-10bp	DUF6453 family protein
066039	fig|066039.3.peg.81	48490	48032	-	3Base/5BMM	13-15bp	Phage tail fiber protein*
066039	fig|066039.3.peg.82	48568	51579	-	AGGAG	5-10bp	Phage tail fiber protein

Genome sequences were annotated using Prokka v1.12, RAST, and were manually curated using BLASTp. Ribosome binding sites (RBS) of genes were identified using Prodigal v2.6.3 (https://github.com/hyattpd/Prodigal).

*fig|066039.3.peg.81 encodes phage tail fiber protein, which is predict to be a depolymerase.

Supplementary Table 7
Genome characterization of *Webervirus* genus phages

Webervirus genus phage	Accession no.	Size (Kb)	GC%	Protein	tRNAs
Klebsiella phage KP36	JF501022.1	49.8	51	79	0
Klebsiella virus GML-KpCol1	NC_047907.1	50.25	51	78	0
Klebsiella phage KPN N141	NC_047841.1	49.09	51	76	0
Klebsiella phage NJS2	NC_048043.1	50.13	51	79	0
Klebsiella phage KpKT21phi1	NC_048143.1	49.11	51	76	0
Klebsiella phage NJR15	NC_048044.1	49.47	51	75	0
Klebsiella phage GH-K3	NC_048162.1	49.43	50	77	0
Klebsiella phage TAH8	NC_048042.1	49.34	51	76	0
Klebsiella phage TSK1	NC_048126.1	49.86	51	74	0
Klebsiella phage 1513	KP658157.1	49.46	51	72	0
Klebsiella phage Sushi	NC_028774.1	48.75	51	76	0
Klebsiella phage MezzoGao	NC_047850.1	49.81	51	76	0
Klebsiella phage NJS1	NC_048024.1	49.29	51	71	0
Klebsiella phage KLPN1	KR262148.1	49.04	51	72	0
Klebsiella phage PKP126	KR269719.1	50.93	51	78	0
Klebsiella phage vB_KpnS_KpV522	NC_047784.1	51.1	51	79	0
Klebsiella phage KOX1	NC_047825.1	50.53	51	81	0
**Klebsiella phage 066039**	MW042802.1	51.63	52	81	0
Enterobacter virus F20	NC_043469.1	51.54	48	83	0

Supplementary Table 8

Genome Comparation between *Webervirus* genus phages and phage P39 (066039) using blastn

Description	Name	Coverage, %	Identity, %	Overall DNA sequence homolog (coverage× identity, %)	Accession
**Klebsiella phage 066039, complete genome**	Klebsiella phage 066039	100	100	100	MW042802.1
Klebsiella phage vB_KpnS_KpV522, complete genome	Klebsiella phage vB_KpnS_KpV522	87	97.1	84.477	NC_047784.1
Klebsiella phage KOX1, complete genome	Klebsiella phage KOX1	87	96.62	84.0594	NC_047825.1
Klebsiella phage PKP126, complete genome	Klebsiella phage PKP126	85	95.26	80.971	KR269719.1
Klebsiella phage KLPN1, complete genome	Klebsiella phage KLPN1	82	92.61	75.9402	KR262148.1
Enterobacteria phage F20, partial genome	Enterobacter virus F20	88	83.9	73.832	NC_043469.1
Klebsiella phage NJR15, complete genome	Klebsiella phage NJR15	88	83.87	73.8056	NC_048044.1
Klebsiella phage TAH8, complete genome	Klebsiella phage TAH8	88	83.74	73.6912	NC_048042.1
Klebsiella phage NJS2, complete genome	Klebsiella phage NJS2	86	84.27	72.4722	NC_048043.1
Klebsiella phage KPN N141, complete genome	Klebsiella phage KPN N141	85	83.82	71.247	NC_047841.1
Klebsiella phage NJS1, complete genome	Klebsiella phage NJS1	85	83.73	71.1705	NC_048024.1
Klebsiella phage MezzoGao, complete genome	Klebsiella phage MezzoGao	84	83.92	70.4928	NC_047850.1
Klebsiella phage KP36, complete genome	Klebsiella phage KP36	83	83.68	69.4544	JF501022.1
Klebsiella phage KpKT21phi1, complete genome	Klebsiella phage KpKT21phi1	83	83.6	69.388	NC_048143.1
Klebsiella phage Sushi, complete genome	Klebsiella phage Sushi	82	83.91	68.8062	NC_028774.1
Klebsiella virus GML-KpCol1, complete genome	Klebsiella virus GML-KpCol1	82	83.87	68.7734	NC_047907.1
Klebsiella phage GH-K3, complete genome	Klebsiella phage GH-K3	80	84.08	67.264	NC_048162.1
Klebsiella phage 1513, complete genome	Klebsiella phage 1513	80	83.95	67.16	KP658157.1
Klebsiella phage TSK1, complete genome	Klebsiella phage TSK1	79	83.69	66.1151	NC_048126.1

Supplementary Table 9
SNPs between the genome sequence of B2-1 and its P39 resistant mutants using snippy

	contig no.	Position	Type	Reference	Alteration	Region	Strand	Effect	Locus_tag	Gene	Product
B2-1	00014	84867	snp	G	A	non-coding regions					
	00032	45406	snp	A	G	CDS	+	missense_variant c.346A>G p.Ile116Val	HIOACJJI_04689	*traS*	Protein TraS
	00043	11102	snp	C	T	CDS	+	stop_gained c.532C>T p.Gln178*	HIOACJJI_05113	*epsJ*	hypothetical protein
	00048	96	snp	G	T	CDS	+	missense_variant c.63G>T p.Gln21His	HIOACJJI_05221		hypothetical protein
	00048	626	snp	T	C	CDS	+	missense_variant c.593T>C p.Leu198Pro	HIOACJJI_05221		hypothetical protein
	00048	1974	snp	A	G	CDS	+	synonymous_variant c.51A>G p.Pro17Pro	HIOACJJI_05224		hypothetical protein
	00055	3043	complex	AAGA	GAAC	non-coding regions					
	00098	96	snp	A	T	non-coding regions					
B2-2	00012	51170	snp	C	A	CDS	-	missense_variant c.208G>T p.Asp70Tyr	HOMDKAIB_02591	*pdeC*	putative cyclic di-GMP phosphodiesterase PdeC
	00014	56644	snp	C	T	CDS	-	missense_variant c.181G>A p.Ala61Thr	HOMDKAIB_02836	*hfq*	RNA-binding protein Hfq
	00029	60067	snp	C	T	CDS	-	missense_variant c.713G>A p.Arg238Gln	HOMDKAIB_04201		IS5 family transposase IS903
	00061	7957	snp	A	G	non-coding regions					
	00061	8005	snp	A	G	non-coding regions					
	00062	10729	snp	T	C	non-coding regions					
	00068	2833	snp	T	G	CDS	-	missense_variant c.46A>C p.Ser16Arg	HOMDKAIB_05479		hypothetical protein
	00143	51	snp	C	T	non-coding regions					
B2-3	00014	84867	snp	G	A	non-coding regions					
	00055	3043	complex	AAGA	GAAC	non-coding regions					

Supplementary Table 10
Sanger sequencing results of *wcaJ* PCR amplicons of P24-resistant mutants isolated *in vivo*

Mutants	Insert sequence	Insertion nucleotide position (numbered from the start codon)	IS Family	DR (Direct repeat)
W1	IS*903B*	1207	IS*5*	-
W2	IS*Kpn26*	627	IS*5*	CTAA
W3	IS*Kpn14*	1238	IS*1*	-
W4	IS*Kpn14*	1238	IS*1*	-
W5	IS*903B*	295	IS*5*	-
W6	IS*903B*	295	IS*5*	-
W7	IS*903B*	1207	IS*5*	-
W8	IS*Kpn26*	509	IS*5*	TTAG
W9	IS*Kpn26*	902	IS*5*	TTAA
W10	IS*903B*	1196	IS*5*	-
W11	IS*Kpn26*	1146	IS*5*	CTAG
W12	IS*903B*	1207	IS*5*	-
W13	IS*Kpn14*	1238	IS*1*	-
W14	IS*Kpn26*	1146	IS*5*	CTAG
W15	IS*Kpn26*	936	IS*5*	TTAG
W16	IS*Kpn26*	413	IS*5*	CTAA
W17	IS*Kpn26*	936	IS*5*	TTAG
W18	IS*903B*	275	IS*5*	-
W19	IS*Kpn26*	690	IS*5*	CTAA
W20	IS*Kpn26*	1361	IS*5*	CTAA

Supplementary Table 11
SNPs between the ST11 CRKP strains using in this study to determine phage host ranges.

Snp*-dists 0.7.0	015134	015318	015581	015584	015712	015785	020003	020030	020037	020046	020058	020143	090527
015134 (B0)	0	55	224	59	72	11	16	83	46	165	72	66	72
015318	55	0	242	16	90	61	57	100	62	182	91	85	89
015581	224	242	0	246	221	231	227	230	234	89	221	213	257
015584	59	16	246	0	93	65	61	104	65	186	95	89	93
015712	72	90	221	93	0	78	73	66	82	163	67	47	104
015785	11	61	231	65	78	0	23	89	53	169	78	70	79
020003	16	57	227	61	73	23	0	85	48	167	74	67	74
020030	83	100	230	104	66	89	85	0	93	171	77	61	115
020037	46	62	234	65	82	53	48	93	0	175	82	76	82
020046	165	182	89	186	163	169	167	171	175	0	163	159	197
020058	72	91	221	95	67	78	74	77	82	163	0	59	104
020143	66	85	213	89	47	70	67	61	76	159	59	0	98
090527	72	89	257	93	104	79	74	115	82	197	104	98	0

*SNPs between the genome sequence of ST11 CRKP were identified using Snippy v4.6.0 (https://github.com/tseemann/snippy)

Supplementary Table 12
Primers used for amplification and sequencing

Primer	Sequence (5′→3′)
mshA-F-SacI	AACGAGCTCATGAAGAAAACAATATTTG
mshA-R-HindIII	AACAAGCTTTTATTTAAGATTATTGAGA
wcaJ-F-SacI	AACGAGCTCATGAAAACTTTCACGCA
wcaJ-R-HindIII	AACAAGCTTTCAATATGCAGACTTATTAA
epsJ-F-SacI	AACGAGCTCGTTGTCGATCAAACGGTTAT
epsJ-R-EcoRV	AACGATATCTTACATTTTCCTCGTGATTAA
pdeC-F-SacI	AACGAGCTCCTGTCGGATGTCAGCT
pdeC-R-HindIII	AACAAGCTTTCAGATCTCCGCGTG
M13R	GTAAAACGACGGCCAGT
M13F	CAGGAAACAGCTATGACC
dfrA-F	ATGAACTCGGAATCAGTACG
dfrA-R	TTAGCCGTTTCGACG

[a]Restriction sites are underlined.
