# Supplementary material for: Characterization of phage resistance and phages capable of intestinal decolonization of carbapenem-resistant Klebsiella pneumoniae in mice
Source: Commun Biol. 2022 Jan 13;5:48. doi: 10.1038/s42003-022-03001-y (PMC8758719; doi:10.1038/s42003-022-03001-y)
Supplement: Supplementary file 2 — Description of Additional Supplementary Files [file 42003_2022_3001_MOESM2_ESM.pdf]

Description of Additional Supplementary Files

File name: Supplementary Data 1

Description: Source results of Fig. 2.

File name: Supplementary Data 2

Description: Source results of Fig. 4.

File name: Supplementary Data 3

Description: Source results of Fig. 8.

File name: Supplementary Data 4

Description: Source results of Supplementary Figure 1.
